# Preparing Medical Students for Anti-racism at the Bedside: Teaching Skills to Mitigate Racism and Bias in Clinical Encounters

**DOI:** 10.15766/mep_2374-8265.11333

**Published:** 2023-08-10

**Authors:** Catherine Tarleton, Wendy Tong, Emily McNeill, Ahmed Owda, Beth Barron, Hetty Cunningham

**Affiliations:** 1 First-Year Resident, Department of Obstetrics, Gynecology and Women's Health, University of Hawai'i at Mānoa John A. Burns School of Medicine; 2 First-Year Resident, Department of Medicine, McGaw Medical Center of Northwestern University; 3 First-Year Resident, Department of Neurology, University of California, San Francisco Weill Institute for Neurosciences; 4 First-Year Resident, Department of Ophthalmology, University of Michigan Medical School; 5 Associate Professor, Department of Medicine, Columbia University Irving Medical Center; 6 Associate Professor, Division of Child and Adolescent Health, Department of Pediatrics, Columbia University Vagelos College of Physicians and Surgeons, Columbia University Irving Medical Center, and Morgan Stanley Children's Hospital at New York Presbyterian; † Co-last author

**Keywords:** Clinical Teaching/Bedside Teaching, Health Equity, Racism in Medicine, Anti-racism, Diversity, Equity, Inclusion

## Abstract

**Introduction:**

Systemic racism perpetuates health disparities and negatively impacts health care delivery and patient outcomes. Racism and bias can affect every aspect of clinical care, including history-taking, physical examination, laboratory interpretation, note-writing, oral presentation, and decision-making. Medical students must learn racism- and bias-mitigation skills early in their professional development to provide high-quality, equitable care.

**Methods:**

In November 2021, senior medical students and faculty with expertise in promoting health equity and justice in medicine designed and cotaught a Zoom-based, 75-minute, interactive session for second-year medical students. Participants prepared by reading assigned articles. Breakout rooms were used to facilitate small-group discussions. Session topics included use of a structural vulnerability assessment tool, examples of how bias can impact the physical exam, demonstration of how language can transmit bias, and skill practice using neutral instead of stigmatizing language.

**Results:**

Forty second-year medical students participated in the session. Thirty-one students (78%) completed Likert-type surveys evaluating reaction and learning. Results showed improvements in students’ perceptions of their abilities to assess for structural factors that influence health, recognize ways bias can impact clinical encounters, and apply skills to minimize bias in clinical care and decision-making.

**Discussion:**

Providing opportunities for health care learners to think critically about how bias impacts patients and communities and equipping them with tools to begin dismantling exclusionary, racist practices in medicine are achievable and crucial to actualizing a just and equitable health system. This educational session can be adapted for training across health care professions and the educational continuum.

## Educational Objectives

By the end of this session, students will be able to:

1. Describe the purpose of the structural vulnerability assessment and incorporate it into history-taking skills.
2. Identify ways that bias can impact physical exam findings.
3. Recognize why using a race-based equation to measure estimated glomerular filtration rate (eGFR) could adversely impact a patient's care and describe an alternative method of calculating eGFR that does not include race.
4. Explain how aspects of a patient's identity (e.g., race, gender) may affect utilization of interventional cardiac procedures (e.g., cardiac catheterization) and describe possible steps to promote equity in clinical decision-making and access.
5. Explain how the language (including patient identifiers) used in presentations and note-writing can transmit bias.
6. Practice utilizing examples of neutral language instead of stigmatizing language.

## Introduction

Systemic racism and bias are pervasive in medicine and cause increased morbidity and mortality for minoritized populations, perpetuating health disparities.^1^ As movements for racial and social justice have gained visibility and momentum in recent years, there is growing awareness that the traditional focus on individualistic care is insufficient to combat deeply entrenched systemic forces that have concretized health inequities. In response, some medical schools have initiated anti-racism efforts, including curricular innovations that aim to shift medical cultures and systems toward anti-racist change. Students and house staff have frequently been at the forefront of advocacy for anti-racist structural and curricular innovations in medical education.^2^

There is a growing field of scholarship on anti-racism in medical education. A review of publications found that curricular innovations have focused on (a) knowledge-building in the areas of cultural competency, implicit bias, racism, health disparities, and social determinants of health^3^; (b) skill-building in microaggression response, emphasizing speaking up against and encouraging dialogue around microaggressions^4–8^; (c) the use of neutral instead of stigmatizing language to reduce bias transmission when communicating about patients^9–11^; and (d) structural competency in medical education.^12^ Learner populations have included medical students, residents, fellows, faculty, and institutional leaders.

However, there is a lack of educational initiatives designed to impart a cohesive view of multiple concrete clinical skills that combat racism and bias in patient care. Developing anti-racist clinical skills is critical to eliminating health inequities because racism and bias can negatively influence every aspect of clinical care, including history-taking, the physical exam, laboratory interpretation, note-writing, oral presentations, and decision-making. To address this gap, senior medical students and faculty educators developed, piloted, and evaluated a workshop to teach second-year medical students concrete skills promoting equitable care at multiple points during a clinical encounter.

Our session is unique in that it applies multiple concepts and skills aimed at mitigating bias in patient care, organizing them around a typical patient encounter. In addition, our session is designed for preclinical medical students at a critical time during training before they begin their clinical rotations. Our session is also uniquely proactive by having participants learn and practice bias-mitigation skills. We chose this action-oriented, case-based approach that focuses on skills development because eliminating racism and inequity in medicine requires training future health care providers to actively apply tools to uproot deeply entrenched racism and bias.

## Methods

### Overview

To increase knowledge of how racism impacts patient care and teach bias-mitigation skills, we designed a 75-minute workshop entitled Preparing Medical Students for Anti-racism at the Bedside (Appendix A) that would be appropriate for all preclinical medical students prior to beginning their clinical rotations. We divided the session into three small-group activities to enhance student engagement. We introduced each step of a clinical encounter, highlighted specific skills that could be applied to increase equity and decrease bias, and provided an opportunity to practice some of these skills.

### Implementation

#### Curricular placement

We offered the workshop to second-year medical students as part of the preclinical course teaching history-taking and physical exam skills. The session occurred several weeks prior to students’ entry into clinical clerkships and was one of three offered options—anti-racism at the bedside, clinical dermatology, and surgical skills—all of which were designed to be immediately applicable in the clinical setting. All participants had previously completed anti-racism coursework, including an 8-hour prematriculation reading assignment and small-group discussion curriculum focused on texts about historic and current systemic racism, bias, privilege, health equity, genetics, and health disparities.

#### Facilitation

One faculty educator and one to two fourth-year medical students from the medical school's Equity and Justice (E&J) Fellowship^13^ facilitated each workshop. E&J fellows were students selected for their exceptional interest and activism regarding anti-racism in medical education. Faculty facilitators, who had heterogeneous health equity knowledge levels, participated in a 1-hour preparatory training with E&J fellows to review the workshop and discuss content-related questions. In addition, student–faculty cofacilitator dyads met separately to coordinate logistics. Facilitators were encouraged to review the facilitation guide (Appendix B) and all assigned articles (prework below).

#### Prework

Student prereadings^14–16^ informed small-group discussions. All students read (a) a short article about racial bias in pulse oximetry measurements^14^ and (b) either an article advocating replacement of estimated glomerular filtration rate (eGFR) race-based equations with equations omitting race and including creatinine and cystatin C^15^ or an article highlighting racial bias in utilization of interventional cardiac procedures.^16^ Students summarized the main points of the second paper they read for their peers. Dividing prereadings fostered peer teaching and minimized student preparation workload.

#### Session agenda

The session agenda content can be found in Appendix A. At the beginning of the session, facilitators provided students with a patient one-liner and encouraged them to consider salient social history questions to engage them early and introduce the centrality of social determinants of health. Facilitators then introduced the structural vulnerability assessment (Appendix C),^17^ a clinical tool designed to aid provider identification of structural contributors (e.g., housing, food access, legal status, education) to health and disease. Faculty facilitators provided additional suggestions based on their own clinical experience for how to assess for and overcome structural barriers to care.

After this initial section on history-taking, the next section focused on the physical exam. Students discussed ways in which historical and current systemic biases could impact practitioners’ approach to the physical examination. In particular, the session focused on the dermatological exam and introduced students to an optional resource for diagnosing dermatological findings on skin of color: VisualDx.^18^ Additionally, students discussed disparities in detection of hypoxemia on skin of color, centering on the prereading about racial bias in pulse oximetry measurements.^14^ Facilitators asked students to brainstorm solutions they might utilize in a clinical environment using the information and data presented.

The next part of the workshop was about clinical decision-making. In breakout groups, students discussed the article they had read on either eGFR^15^ or interventional cardiology disparities.^16^ Again, groups pondered anti-racist clinical solutions to promote health equity and eliminate health disparities.

The session ended with discussion and skills practice concerning oral presentations and note-writing. Using guidelines from the University of Washington,^19^ students considered the use of patient identifiers in presentations and notes. They then focused specifically on language and the transmission of bias using a 2018 study^11^ that looked at stigmatizing versus neutral language in a clinical vignette about a patient with sickle cell disease. The study found that stigmatizing language was associated with more negative attitudes toward the patient and less aggressive pain management. Students were given the clinical vignette with stigmatizing language and wrote a corrected version using neutral language in small groups.

### Evaluation

We created a retrospective pre/post survey utilizing Likert-type questions based on the learning objectives to measure participants’ reaction to and learning from the session (Appendix D). With the retrospective pre/post survey, students were asked once at the end of the session to evaluate their self-perceived knowledge and abilities both before and after the session. We chose this evaluation method to increase response rate and avoid the confounding factors associated with pretests, including students not knowing what they do not know and therefore reporting a more positive reflection of their knowledge and skills than they would if they better understood what was expected.^20^ Approval for this study was obtained from the Columbia University Medical Center Institutional Review Board.

We analyzed the survey data using a binary dependent variable of agree or disagree for each survey question and 2 × 2 contingency tables. We calculated *p* values using the McNemar statistical test with α = .05. We conducted the analyses in R 4.2.2 (R Foundation for Statistical Computing).

## Results

Forty second-year medical students participated in the workshop. Thirty-one students (78% of participants) completed the retrospective pre/post survey at the end of the session, including 13 (33%) who completed the optional open-ended questions. The survey consisted of 20 Likert-type questions (10 presession and 10 postsession) and two optional open-ended questions.

**Figure 1.**
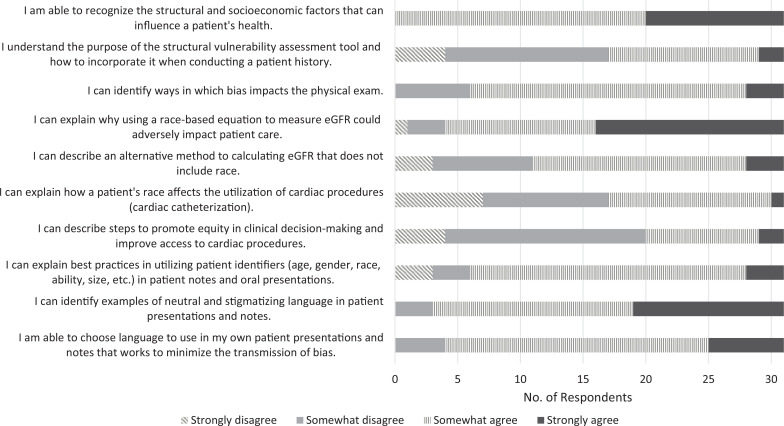
Presurvey results (*N* = 31). Abbreviation: eGFR, estimated glomerular filtration rate.

**Figure 2.**
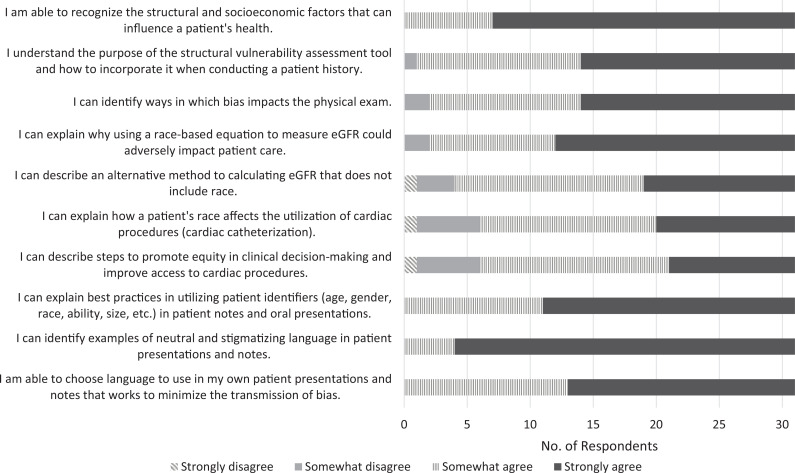
Postsurvey results (*N* = 31). Abbreviation: eGFR, estimated glomerular filtration rate.

Participants indicated that the workshop improved their perceptions of their abilities in all domains evaluated, including recognizing and assessing for structural factors that influence health, recognizing ways bias can affect the physical exam, and applying skills to minimize bias in clinical care and decision-making. All students indicated that after the session, they somewhat or strongly agreed they could recognize the structural and socioeconomic factors that can influence a patient's health, explain best practices in utilizing patient identifiers, identify examples of neutral and stigmatizing language in patient presentations and notes, and choose language in their own presentations and notes to minimize bias transmission (Figures 1 and 2; Table 1). There was a statistically significant increase in respondents’ self-perceived ability to (1) understand and use the structural vulnerability assessment tool (*p* < .001), (2) describe an alternative method for calculating eGFR that does not include race (*p* = .02), (3) explain how a patient's race affects the utilization of cardiac procedures (*p* = .003), (4) describe steps to promote equity in clinical decision-making and improve access to cardiac procedures (*p* < .001), and (5) explain best practices in utilizing patient identifiers in presentations and notes (*p* = .04; Table 1).

**Table 1.**
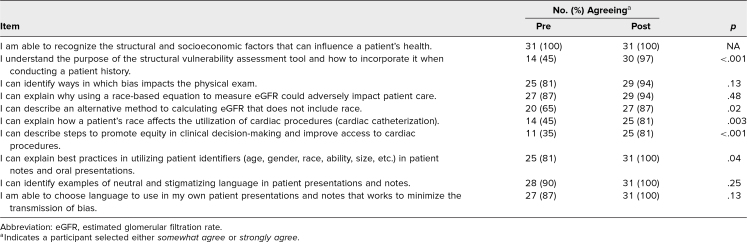
Quantitative Results: Participants Somewhat or Strongly Agreeing on Pre/Post Survey Questions (*N* = 31)

Thirteen participants completed the two open-ended questions. Eleven participants indicated that the note-writing activity and practice replacing stigmatizing with neutral language were most useful. Students also appreciated learning in small groups and identified the presession readings, structural vulnerability assessment tool, and physical exam section as helpful workshop components. Some of the suggestions students made for improvements included clearer instructions regarding prereading assignments, increased time in small groups, and improved integration of session materials with the longitudinal preclinical curriculum (Table 2).

**Table 2.**
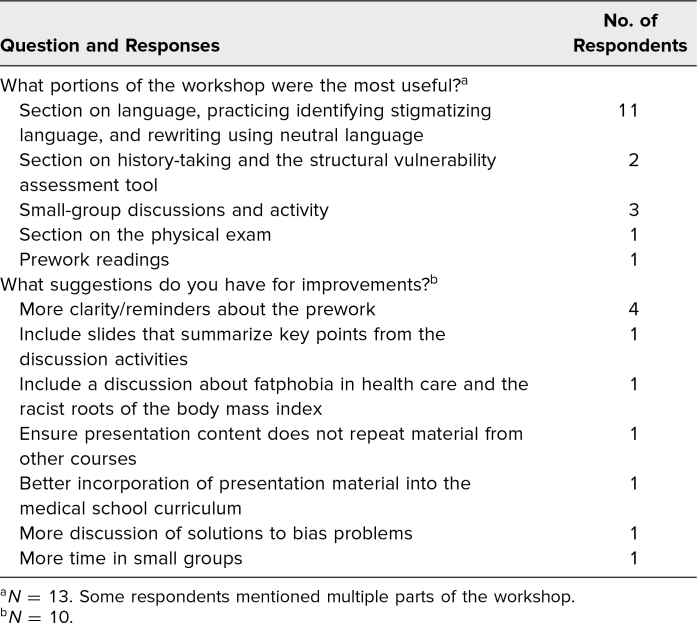
Qualitative Results: Responses to the Optional Free-Response Survey Questions

## Discussion

Our workshop offers medical students concrete, applicable knowledge and skills to recognize and mitigate the effects of historical and systemic racism and bias at multiple points in a typical patient encounter. Survey results indicated that students found the session useful and gained self-perceived applicable knowledge and skills. Due to its success, the session has been incorporated into the required curriculum for all students preparing for the transition to clinical rotations.

Because curricular time is a major challenge for medical education, we worked to condense key concepts from the growing literature on anti-racist clinical skills into a onetime workshop. We sought to optimize curricular time by targeting students who would be in a position to practice these skills the following month. Lessons learned include the need for ongoing curricular mapping of anti-racist content and for collaboration between students and faculty to determine the best ways to integrate anti-racism. To meet this challenge, our medical school created a new longitudinal health equity and justice curricular thread, funded five health equity fellowships for medical students, and hired a health equity thread faculty leader. We predict that these initiatives will facilitate integration and scaffolding of curricula throughout the 4-year program to cohesively teach anti-racist knowledge and skills without unintentional repetition. Another challenge we faced in the development of our workshop was recruiting faculty facilitators with sufficient knowledge to facilitate the session. This highlights the need for faculty development in health equity and anti-racism topics.

Our workshop has a few limitations. Because participants opted into the session, there may have been participation bias given that students who are especially interested in health equity and anti-racism may react more favorably to the session than those who are not. Additionally, the retrospective pre/post survey relied on self-assessment and subjective feelings of increased ability and comfort with the learning objectives rather than direct assessment of knowledge or skill attainment. More objective evaluation methods (e.g., an OSCE or short-answer quiz) could be used to strengthen assessment of learning objectives. Retrospective pre/post surveys are used to avoid response-shift bias and prevent students from overestimating their knowledge before an event. However, they do introduce recall bias (where memory can be distorted in being asked to recall prior knowledge and skill level) and response shift bias (desire to demonstrate growth leading to an underestimation of prior level). Those who wish to adapt our workshop could consider doing both a true pretest and a retrospective pretest to better measure and understand growth.

Educating the next generation of clinicians to provide inclusive care that mitigates the effects of systemic racism and bias in medicine is critical to reducing health disparities that cause exaggerated death and disability within historically marginalized populations. Our workshop addresses some of the most glaring clinical areas that lead to disparity. This session has been incorporated into our medical school's new longitudinal health equity and justice curricular thread and serves to translate preclerkship foundational knowledge into crucial clinical skills. We believe this session would also be a strong addition to educational efforts at the graduate and continuing medical education levels with tailoring toward clinical specialty. In particular, training in modifying stigmatizing language into neutral language that honors patient stories and recognizes social determinants of health is critical and applicable at every level of training and in every medical specialty. Other future directions include incorporating interdisciplinary team members, such as social work and nursing, in educational efforts and skill development around mitigating racism and bias in patient encounters.

## Appendices


Presentation.pptxFacilitation Guide.docxStructural Vulnerability Assessment Tool.docxSurvey Questions.docx
*All appendices are peer reviewed as integral parts of the Original Publication.*
