# Supplementary material for: Preparing Medical Students for Anti-racism at the Bedside: Teaching Skills to Mitigate Racism and Bias in Clinical Encounters
Source: MedEdPORTAL. 2023 Aug 10;19:11333. doi: 10.15766/mep_2374-8265.11333 (PMC10412739; doi:10.15766/mep_2374-8265.11333)
Supplement: Supplementary file 1 — Presentation.pptxFacilitation Guide.docxStructural Vulnerability Assessment Tool.docxSurvey Questions.docx [file mep_2374-8265.11333-s001.zip › B. Facilitation Guide.docx]

**Appendix B: Facilitation Guide**

**Preparation:**

- Review the articles assigned to students for this session:
  - Racial Bias in Pulse Oximetry [1]
  - Black lives matter … in the cath lab, too! A proposal for the interventional cardiology community to counteract bias and racism [2]
  - New Creatinine- and Cystatin C-Based Equations to Estimate GFR without Race [3]
- Meet with your co-facilitator(s) to plan how you will share running the session
- OPTIONAL: review the following articles used in the presentation to learn more about some of the concepts in the session
  - Structural Vulnerability: Operationalizing the Concept to Address Health Disparities in Clinical Care [4]
  - Do Words Matter? Stigmatizing Language and the Transmission of Bias in the Medical Record [5]
- OPTIONAL: review the University of Washington guidelines for using patient identifiers [6]. These are discussed in the last part of the session on patient presentations and note-writing (slides 20-22)
- OPTIONAL: practice using visualDx.com [7] to view dermatological findings on skin of color
  - To find/use visualDx.com:
    - Open UpToDate [8]
    - Search visualDx, then scroll down until you see the option “Launch visualDx” and click on it
    - Once visualDx is open, you can click on “Dermatology all skin types” or “Dermatology skin of color.”
    - You can also use the text box to search specific symptoms or diseases
    - For example, if you search “measles,” there are buttons on the top that allow you to view measles on “all skin types” or “skin of color”
- OPTIONAL: these are additional resources for facilitators to further explore dermatological diagnosis and skin of color if interested
  - Mukwende M, Tamonv P, Turner M (2020). *Mind the Gap: a handbook of clinical signs on black and brown skin*. London: St Georges, University of London. <https://www.blackandbrownskin.co.uk/mindthegap>
  - Kelly AP, Taylor SC, Lim HC, Serrano AMA (2015). *Taylor and Kelly’s Dermatology for Skin of Color*, 2nd edition. New York: McGraw-Hill Medical
  - Jackson-Richards D, Pandya AG (2014) *Dermatology Atlas for Skin of Color*. New York: Springer.
  - Claudia M.Y.A. Donkor, Jeannette Aryee-Boi, Itohan Roseline Osazuwa, Francis Kwame Afflu, Andrew F. Alexis (2021). [Atlas of Dermatological Conditions in Populations of African Ancestry | SpringerLink](https://nam02.safelinks.protection.outlook.com/?url=https%3A%2F%2Flink.springer.com%2Fbook%2F10.1007%2F978-3-030-72617-1&data=05%7C01%7Chc451%40cumc.columbia.edu%7C7a2c9f6b617141b0632108da81d5601f%7Cb0002a9b0017404d97dc3d3bab09be81%7C1%7C0%7C637965050720786518%7CUnknown%7CTWFpbGZsb3d8eyJWIjoiMC4wLjAwMDAiLCJQIjoiV2luMzIiLCJBTiI6Ik1haWwiLCJXVCI6Mn0%3D%7C3000%7C%7C%7C&sdata=Kz0q7SsL%2F25ZORfk6XgGwnLcn2QJa5eIf3UGN7AuC2o%3D&reserved=0)

**Making breakout groups for small group discussions (if on Zoom):**

- Once you login, you’ll need to claim host (click on participants, at the bottom click on claim host, put the key in from the Zoom link)
- Hover over the bottom and you should now see breakout rooms. Click on this, select the number of breakout rooms you would like, and assign manually
- Each time you want to put students in their breakout groups, click breakout rooms and select options – select automatically move students to their rooms, breakout rooms close after. Choose the amount of time for the breakout session and countdown after closing room (30 seconds)

**Power point presentation:**

- Detailed notes for each slide can be found in the slide deck (Appendix A)
- For small group discussions, remember to provide students with discussion questions either through email, giving them hard copies, or if on Zoom, copying the discussion questions into the chat BEFORE sending students off into their small groups
- Below in red are possible responses for small group discussion questions/activities (many points were drawn from the papers themselves)
  - **Small group discussion #1: racial bias in pulse oximetry measurements** (slides 13-15)
    - What surprised you about this study?
    - What may have led to this issue to begin with?
      - Pulse oximeter originally developed in populations that were not racially diverse
    - What could be done to prevent this from happening in the future?
      - Important for research to include diverse populations, otherwise the research findings cannot be generalized to the larger population. Racial bias in pulse ox measurements is an example of what can happen when we do not include diverse populations in research and medical device development.
    - What take home points do you want to remember?
    - What could you do differently in a clinical environment knowing this information?
      - Reminder to consider oxygen saturation as one data point in the context of the larger clinical picture. For example, if you have a patient whose presentation is concerning for hypoxemia but their pulse oximeter reading is not as low as you would expect and they have darker skin, it’s important not to discount your concern for hypoxemia based on the pulse ox reading.
      - Consider getting an ABG if you need an accurate oxygen saturation level.
      - This paper also reminds us that bias is built into the way we practice medicine. We may not know all the ways bias impacts our patients and practice, but we can and should remain critical and open-minded to how bias may be affecting our interactions and how we care for patients.
  - **Small group discussion #2: bias in decision-making tools** (slides 17-18)
    - How does bias affect your assigned topic?
      - eGFR: current equation used to calculate eGFR uses race as a parameter.
      - Interventional cardiology: interventional cardiac procedures are often underutilized in patients of color leading to excess morbidity and mortality
    - What problems exist?
      - eGFR: Current guidelines are to use an eGFR equation that uses creatinine and includes a race variable (Black vs non-Black). Race is a social construct with no biological basis. There is actually more genetic variation among people of the same race than between individuals of different races. There is no inherent biological difference between individuals who are Black and those who are non-Black, making the use of this variable when calculating eGFR problematic. In fact, the race-based eGFR equation was found to overestimate GFR in Black people. When race adjustment was omitted, measured GFR was under-estimated in Black people. Over-estimation of GFR leads to underdiagnosis of CKD, inadequate treatment, and increased risk of progression to ESRD. Black people are disproportionately affected by ESRD, but also have lower rates of kidney transplant. We also do not want to underestimate GFR because this could lead to overdiagnosis of CKD and missed opportunities for therapies contraindicated at a low GFR.
      - Interventional cardiology: A 1999 New England Journal of Medicine paper found that women and Black people are both statistically significantly less likely to be referred for cardiac catheterization than men and white people, respectively [9]. Barriers and disparities still exist today in access to high-quality acute cardiac hospitals, and also disparities in provider decisions about which patients will undergo interventional cardiac procedures and the utilization of these procedures.
    - What led to this issue?
      - eGFR: Systemic racism underlying false notions that there is a biological difference between individuals of different races. There were also previous studies that indicated a higher average serum creatinine level for the same measured GFR level in Black participants.
      - Interventional cardiology: Systemic racism influencing the healthcare experiences of Black patients. Research has found that Black patients have longer wait times in the ED, longer wait times for angiography, preferential use of bare metal vs drug-eluting stents, and lower likelihood of participating in cardiac rehab on discharge.
    - How could we prevent similar problems in the future?
      - eGFR: 1) be mindful/critical of research subgroup analyses by race and the conclusions that are drawn, 2) be mindful/critical of how race is used in clinical decision-making.
      - Interventional cardiology: 1) quality improvement programs that review program-specific data for racial differences in treatment of ACS and utilization of catheterization lab therapies and interventions to address identified disparities, 2) increase referral rates for cardiac rehab for Black patients (automatic electronic order AND discussion with patient is most effective), 3) increase proportion of Black patients discharged with guideline-directed meds (programs that supply meds to patients on day of discharge recommended), 4) increase patient diversity in interventional cardiac clinical research, 5) enhance consistent community outreach, 6) implicit bias training for catheterization lab professionals, 7) enhance diversity in interventional cardiology (<3% of cardiologists are Black, with smaller numbers for interventional specialists), 8) programs to self-assess via an anti-bias “report card.”
    - What could be done to minimize the effects of bias for this issue?
      - eGFR: 1) Use new eGFR equation that is not race-based. 2) The equation that uses creatinine and cystatin-C and does not include race as a parameter was found to be most accurate at calculating GFR for Black and non-Black participants, minimizing differences in eGFR between race groups and differences in estimated CKD prevalence. 3) Cystatin-C is still not in widespread use for calculating GFR, so some institutions may be using creatinine-based equations without the race variable.
      - Interventional cardiology: see response to above question.
  - **Small group activity: changing stigmatizing to neutral language** (slides 25-26)
    - Below are suggestions from the paper of how to correct stigmatizing language and the reason the original language was stigmatizing
      - Man with SCD (neutral) vs. sickle cell patient (stigmatizing)
        - Equating patient with disease
      - Comes with…10/10 pain (neutral) vs. stating he has 10/10 pain “all up in my arms and legs” (stigmatizing)
        - Casting doubt
      - Typically requires opioid pain meds (neutral) vs. narcotic dependent (stigmatizing)
        - Perpetuating negative stereotypes
      - Has about 8-10 pain crises per year (neutral) vs. in our ED frequently (stigmatizing)
        - Perpetuating negative stereotypes
      - At home he takes 100mg oxycontin (neutral) vs. at home he reportedly takes 100mg oxycontin (stigmatizing)
        - Casting doubt
      - He moved to a new apartment (neutral) vs. housing authority moved him (stigmatizing)
        - Unnecessary SES indicator
      - Spent yesterday afternoon with friends (neutral) vs. hanging out with friends outside McDonalds (stigmatizing)
        - Negative stereotypes, unnecessary SES indicator
      - Recent stress (neutral) vs. some “stressful situations” (stigmatizing)
        - Casting doubt, negative stereotypes
      - Not alleviated by his home pain med regimen (neutral) vs. has not been helped by any of the narcotic meds he says he has already taken (stigmatizing)
        - Casting doubt, negative stereotypes

**Additional questions and challenges:**

Below are some additional questions and challenges for facilitators to think about in preparation for the session, and also to reflect on as we continue anti-racism work beyond this workshop.

1. It is discouraging to know about all the ways bias can adversely impact our patients. How do you keep from losing faith in the medical profession?
2. Are there other tools, besides those presented in this workshop, that you personally use to practice anti-racism in your clinical encounters and settings?
3. How can we balance the concepts of race, ancestry, and genetics? Because there can be some overlap in these.
4. What should we do if we encounter bias in our learning environment? Are there avenues to provide feedback or open discussions about this?

**References**

[1] Sjoding MW, Dickson RP, Iwashyna TJ, Gay SE, Valley TS. Racial Bias in Pulse Oximetry Measurement. *N Engl J Med*. 2020;383(25):2477-2478. doi:[10.1056/NEJMc2029240](https://doi.org/10.1056/NEJMc2029240)

[2] Uzendu AI, Boudoulas KD, Capers Q. Black lives matter … in the cath lab, too! A proposal for the interventional cardiology community to counteract bias and racism. *Catheter Cardiovasc Interv*. 2022;99(2):213-218. doi:[10.1002/ccd.29751](https://doi.org/10.1002/ccd.29751)

[3] Inker LA, Eneanya ND, Coresh J, et al. New Creatinine- and Cystatin C-Based Equations to Estimate GFR without Race. *N Engl J Med*. 2021;385(19):1737-1749. doi:[10.1056/NEJMoa2102953](https://doi.org/10.1056/NEJMoa2102953)

[4] Bourgois P, Holmes SM, Sue K, Quesada J. Structural Vulnerability: Operationalizing the Concept to Address Health Disparities in Clinical Care. *Acad Med*. 2017;92(3):299-307. doi:[10.1097/ACM.0000000000001294](https://doi.org/10.1097/ACM.0000000000001294)

[5] P Goddu A, O’Conor KJ, Lanzkron S, et al. Do Words Matter? Stigmatizing Language and the Transmission of Bias in the Medical Record. *J Gen Intern Med*. 2018;33(5):685-691. doi:[10.1007/s11606-017-4289-2](https://doi.org/10.1007/s11606-017-4289-2)

[6] University of Washington School of Medicine. Patient Identifiers Practice Guidelines. 2020. <https://clime.washington.edu/uwsom-use-of-patient-identifiers-in-foundations-curriculum/>

[7] VisualDx.com. Accessed on 8 August 2022.<https://www.visualdx.com/visualdx/7/?pt=UTD_89238&tk=r0ewgW5lXPStSYYr1PsHQxGtjcTdShGmBnNOw%2BZkhUs%3D>; 2022.

[8] UpToDate.com. Accessed on 8 August 2022. https://www.uptodate.com/contents/search; 2022.

[9] Schulman KA, Berlin JA, Harless W, et al. The effect of race and sex on physicians' recommendations for cardiac catheterization [published correction appears in N Engl J Med 1999 Apr 8;340(14):1130]. *N Engl J Med*. 1999;340(8):618-626. doi:10.1056/NEJM199902253400806
