# Supplementary material for: Preparing Medical Students for Anti-racism at the Bedside: Teaching Skills to Mitigate Racism and Bias in Clinical Encounters
Source: MedEdPORTAL. 2023 Aug 10;19:11333. doi: 10.15766/mep_2374-8265.11333 (PMC10412739; doi:10.15766/mep_2374-8265.11333)
Supplement: Supplementary file 1 — Presentation.pptxFacilitation Guide.docxStructural Vulnerability Assessment Tool.docxSurvey Questions.docx [file mep_2374-8265.11333-s001.zip › C. Structural Vulnerability Assessment Tool.docx]

**Appendix C: Structural Vulnerability Assessment Tool**

*Adapted by the authors from the original paper^[[1]](#endnote-1)^

Financial security:

- Do you have enough money to live comfortably (e.g. pay rent, buy groceries, pay for utilities and phone)?
- What is your primary source of income?

Residence:

- Do you have a safe, reliable place to sleep and store your possessions?

Environment:

- Where do you spend your time each day?
- Do those places feel safe and healthy?

Food access:

- What do you usually eat every day?
- Are you able to access healthy foods like fresh fruits and vegetables?

Social support:

- Do you have friends, family, or other people you can turn to if you need help?
- Do you have a primary care doctor?

Legal status:

- We take care of a lot of people here who do not have papers. Is this a problem you also have?

Education:

- Is reading something that is comfortable for you?
- What is the highest grade level you reached in school?

Discrimination:

- Some people experience discrimination based on various aspects of their identity like their appearance, skin color, accent, gender, sexual orientation, where they are from, religion, how much money they have, etc. If you are comfortable sharing, do you feel you have experienced discrimination before?

Bias: [Questions to ask yourself]

- Is it possible that aspects of this patient’s identity may elicit stigma, negative attitudes, or biases from providers (myself included) that negatively impact the care this patient receives or their experience with the healthcare system?
- What can I do to ensure I do not allow my own biases to influence the way I treat or care for this patient?

**References**

1. Bourgois P, Holmes SM, Sue K, Quesada J. Structural Vulnerability: Operationalizing the Concept to Address Health Disparities in Clinical Care. *Acad Med*. 2017;92(3):299-307. doi:10.1097/ACM.0000000000001294 [↑](#endnote-ref-1)
