# Supplementary material for: Preparing Medical Students for Anti-racism at the Bedside: Teaching Skills to Mitigate Racism and Bias in Clinical Encounters
Source: MedEdPORTAL. 2023 Aug 10;19:11333. doi: 10.15766/mep_2374-8265.11333 (PMC10412739; doi:10.15766/mep_2374-8265.11333)
Supplement: Supplementary file 1 — Presentation.pptxFacilitation Guide.docxStructural Vulnerability Assessment Tool.docxSurvey Questions.docx [file mep_2374-8265.11333-s001.zip › D. Survey Questions.docx]

**Survey**

Please complete the following survey at the end of the session. You will be asked to reflect on your knowledge and skills before the pre-work and after the session. We will be using your responses to improve the session for future students. Thank you.

Instructions: Please indicate with an “X” the appropriate box for each question that reflects your self-perceived knowledge and skills before the pre-work and after the session. The last two questions are optional free-response questions.

|  | Strongly disagree | Somewhat disagree | Somewhat agree | Strongly agree |
| --- | --- | --- | --- | --- |
| 1. BEFORE the pre-work: I was able to recognize the structural and socio-economic factors that can influence a patient's health. |  |  |  |  |
| 2. AFTER the session: I am able to recognize the structural and socio-economic factors that can influence a patient's health. |  |  |  |  |
| 3. BEFORE the pre-work: I understood the purpose of the structural vulnerability assessment tool and how to incorporate it when conducting a patient history. |  |  |  |  |
| 4. AFTER the session: I understand the purpose of the structural vulnerability assessment tool and how to incorporate it when conducting a patient history. |  |  |  |  |
| 5. BEFORE the pre-work: I could identify ways in which bias impacts the physical exam. |  |  |  |  |
| 6. AFTER the session: I can identify ways in which bias impacts the physical exam. |  |  |  |  |
| 7. BEFORE the pre-work: I could explain why using a race-based equation to measure eGFR could adversely impact patient care. |  |  |  |  |
| 8. AFTER the session: I can explain why using a race-based equation to measure eGFR could adversely impact patient care. |  |  |  |  |
| 9. BEFORE the session: I could describe an alternative method to calculating eGFR that does not include race. |  |  |  |  |
| 10. AFTER the session: I can describe an alternative method to calculating eGFR that does not include race. |  |  |  |  |
| 11. BEFORE the pre-work: I could explain how a patient's race affects the utilization of cardiac procedures (cardiac catheterization). |  |  |  |  |
| 12. AFTER the session: I can explain how a patient's race affects the utilization of cardiac procedures (cardiac catheterization). |  |  |  |  |
| 13. BEFORE the pre-work: I could describe steps to promote equity in clinical decision making and improve access to cardiac procedures. |  |  |  |  |
| 14. AFTER the session: I can describe steps to promote equity in clinical decision making and improve access to cardiac procedures. |  |  |  |  |
| 15. BEFORE the pre-work: I could explain best practices in utilizing patient identifiers (age, gender, race, ability, size, etc) in patient notes and oral presentations. |  |  |  |  |
| 16. AFTER the session: I can explain best practices in utilizing patient identifiers (age, gender, race, ability, size, etc) in patient notes and oral presentations. |  |  |  |  |
| 17. BEFORE the pre-work: I could identify examples of neutral and stigmatizing language in patient presentations and notes. |  |  |  |  |
| 18. AFTER the session: I can identify examples of neutral and stigmatizing language in patient presentations and notes. |  |  |  |  |
| 19. BEFORE the pre-work: I was able to choose language to use in my own patient presentations and notes that works to minimize the transmission of bias. |  |  |  |  |
| 20. AFTER the session: I am able to choose language to use in my own patient presentations and notes that works to minimize the transmission of bias. |  |  |  |  |

21. OPTIONAL: What portions of the workshop were the most useful? (free response)

22. OPTIONAL: What suggestions do you have for improvements? (free response)
