# Supplementary material for: Preparing Medical Students for Anti-racism at the Bedside: Teaching Skills to Mitigate Racism and Bias in Clinical Encounters
Source: MedEdPORTAL. 2023 Aug 10;19:11333. doi: 10.15766/mep_2374-8265.11333 (PMC10412739; doi:10.15766/mep_2374-8265.11333)
Supplement: Supplementary file 1 — Presentation.pptxFacilitation Guide.docxStructural Vulnerability Assessment Tool.docxSurvey Questions.docx [file mep_2374-8265.11333-s001.zip › A. Presentation.pptx]

## Slide 1
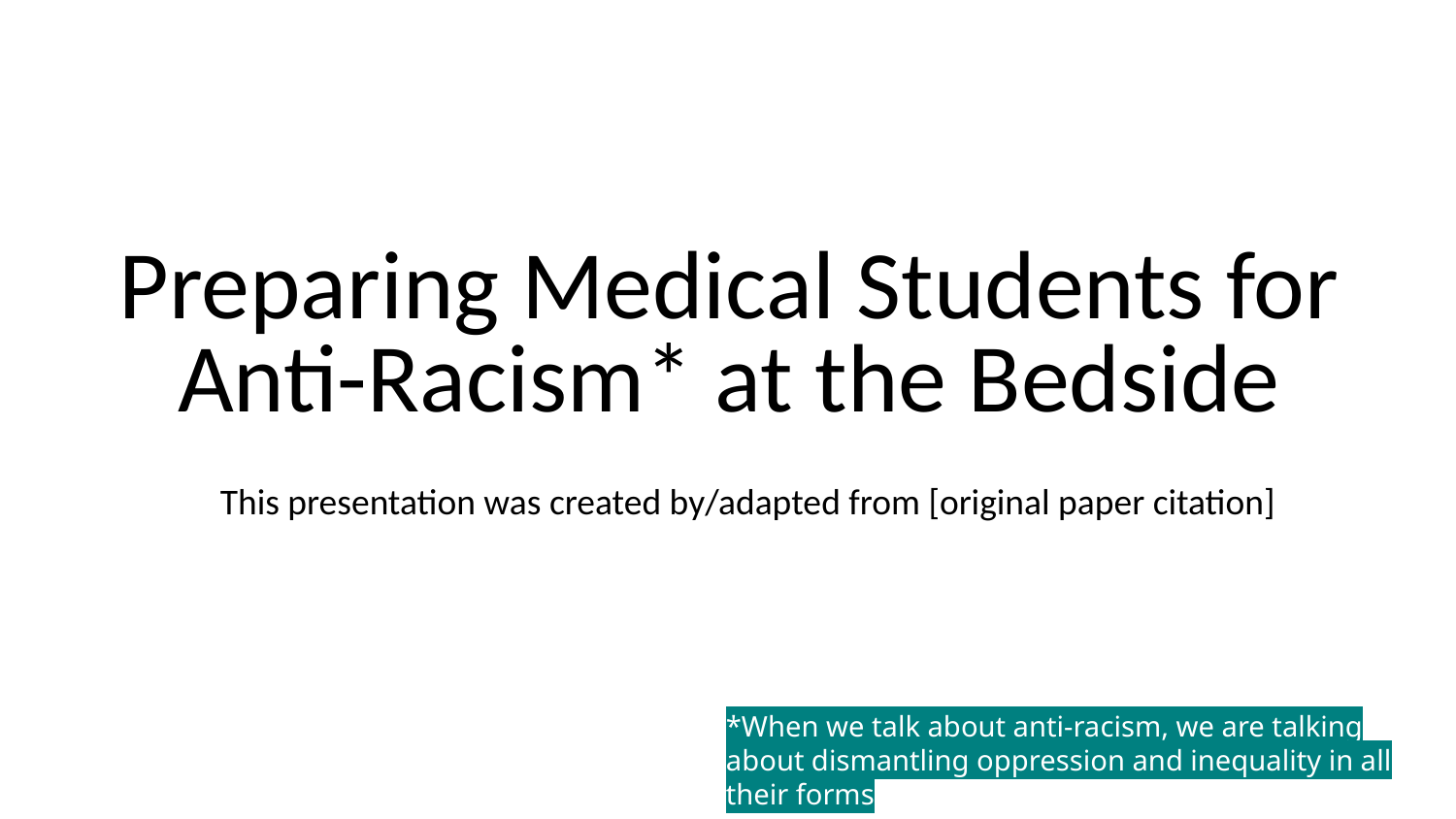

# Preparing Medical Students for Anti-Racism* at the Bedside
This presentation was created by/adapted from [original paper citation]
*When we talk about anti-racism, we are talking about dismantling oppression and inequality in all their forms

## Slide 2
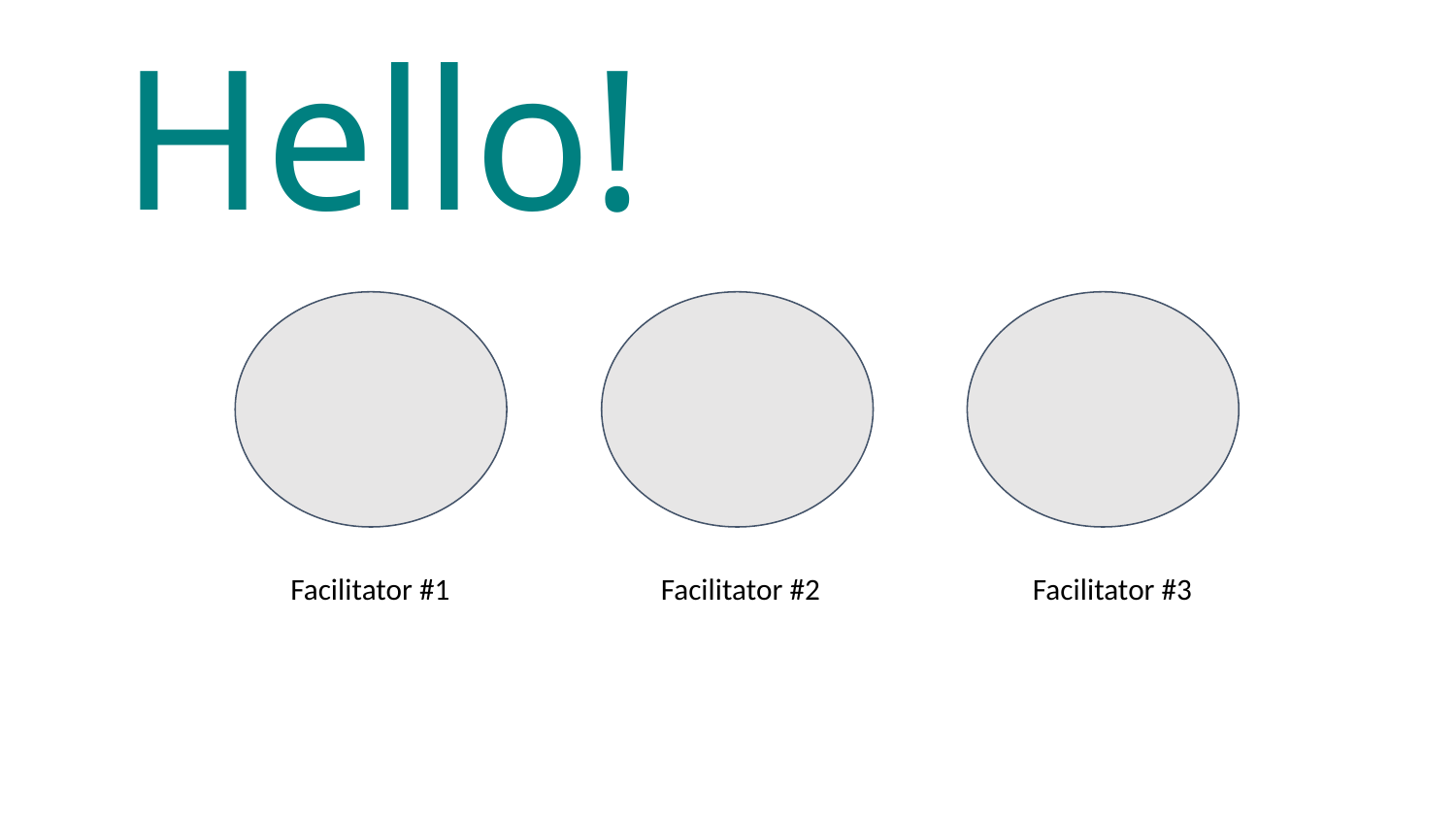

Hello!
Facilitator #1
Facilitator #2
Facilitator #3

## Slide 3
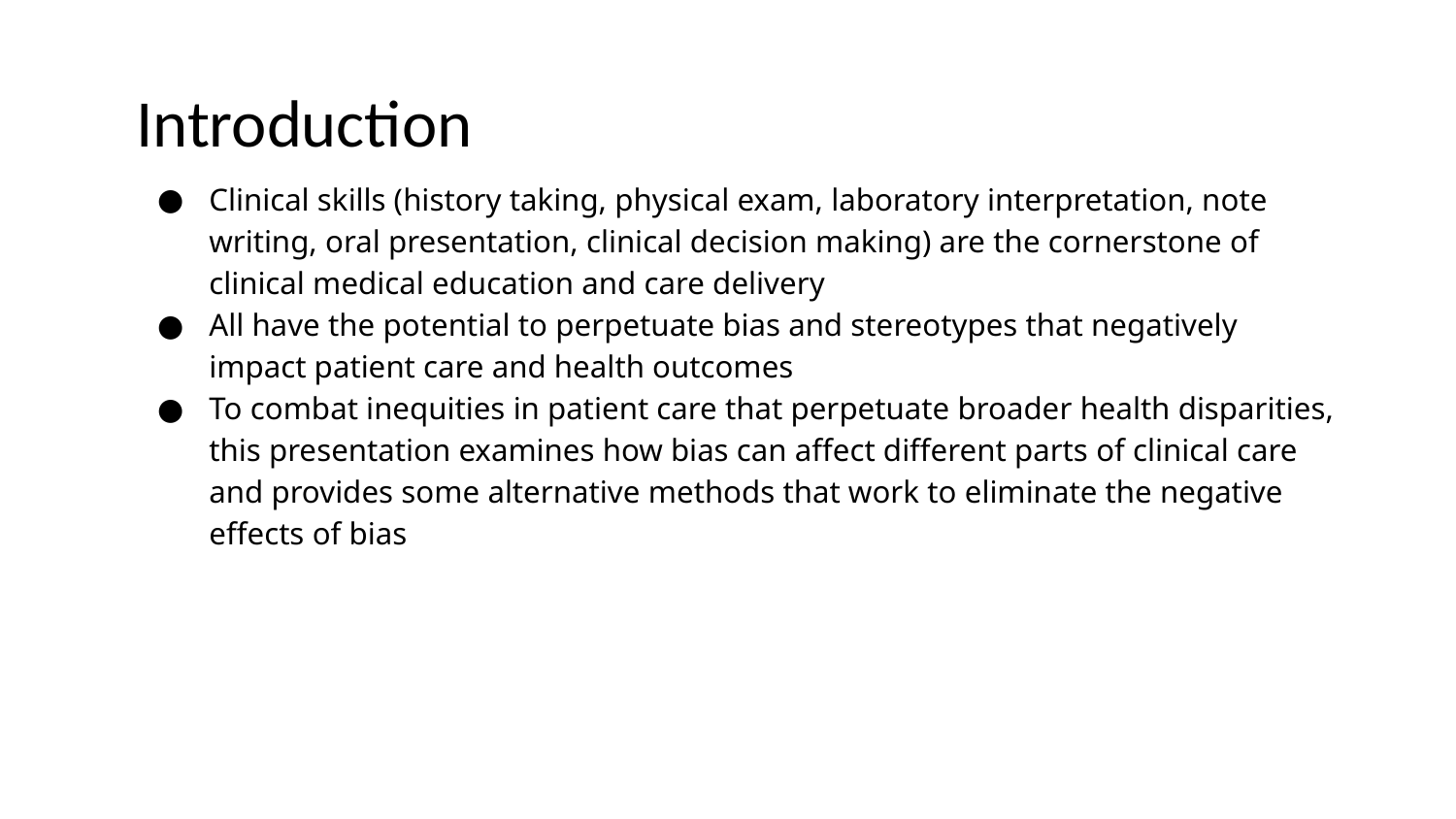

# Introduction
Clinical skills (history taking, physical exam, laboratory interpretation, note writing, oral presentation, clinical decision making) are the cornerstone of clinical medical education and care delivery
All have the potential to perpetuate bias and stereotypes that negatively impact patient care and health outcomes
To combat inequities in patient care that perpetuate broader health disparities, this presentation examines how bias can affect different parts of clinical care and provides some alternative methods that work to eliminate the negative effects of bias

## Slide 4
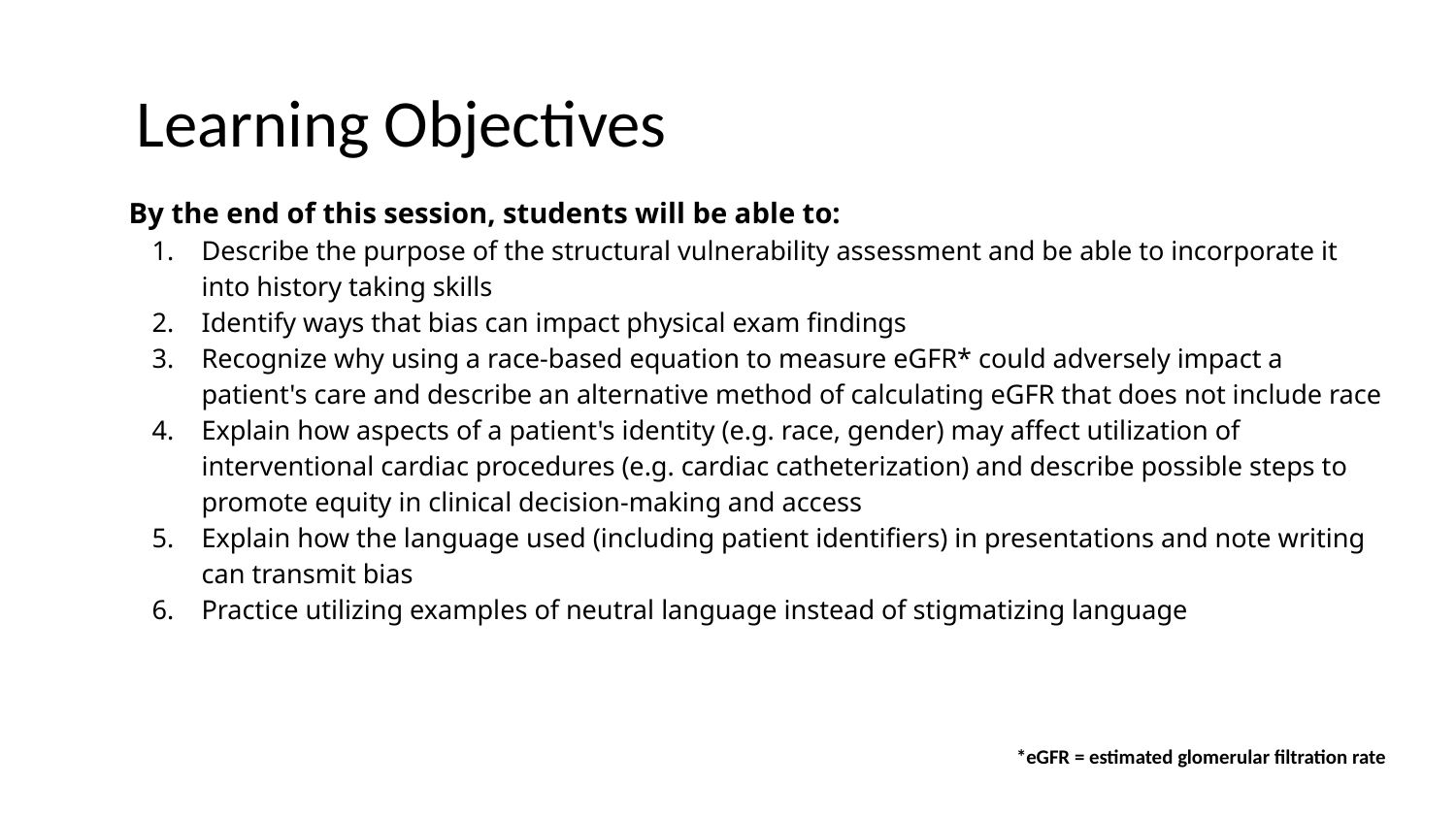

# Learning Objectives
By the end of this session, students will be able to:
Describe the purpose of the structural vulnerability assessment and be able to incorporate it into history taking skills
Identify ways that bias can impact physical exam findings
Recognize why using a race-based equation to measure eGFR* could adversely impact a patient's care and describe an alternative method of calculating eGFR that does not include race
Explain how aspects of a patient's identity (e.g. race, gender) may affect utilization of interventional cardiac procedures (e.g. cardiac catheterization) and describe possible steps to promote equity in clinical decision-making and access
Explain how the language used (including patient identifiers) in presentations and note writing can transmit bias
Practice utilizing examples of neutral language instead of stigmatizing language
*eGFR = estimated glomerular filtration rate

## Slide 5
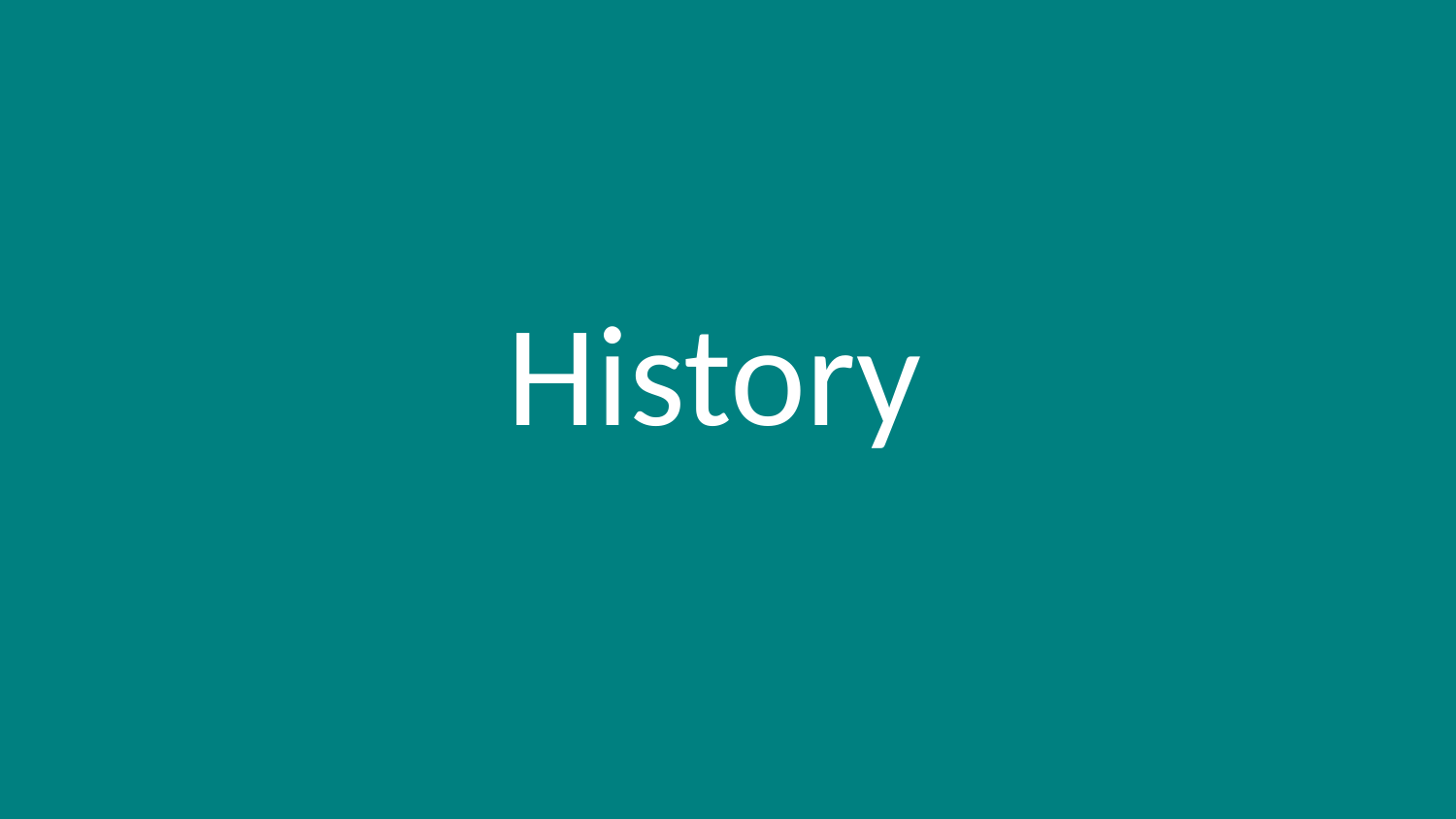

# History

## Slide 6
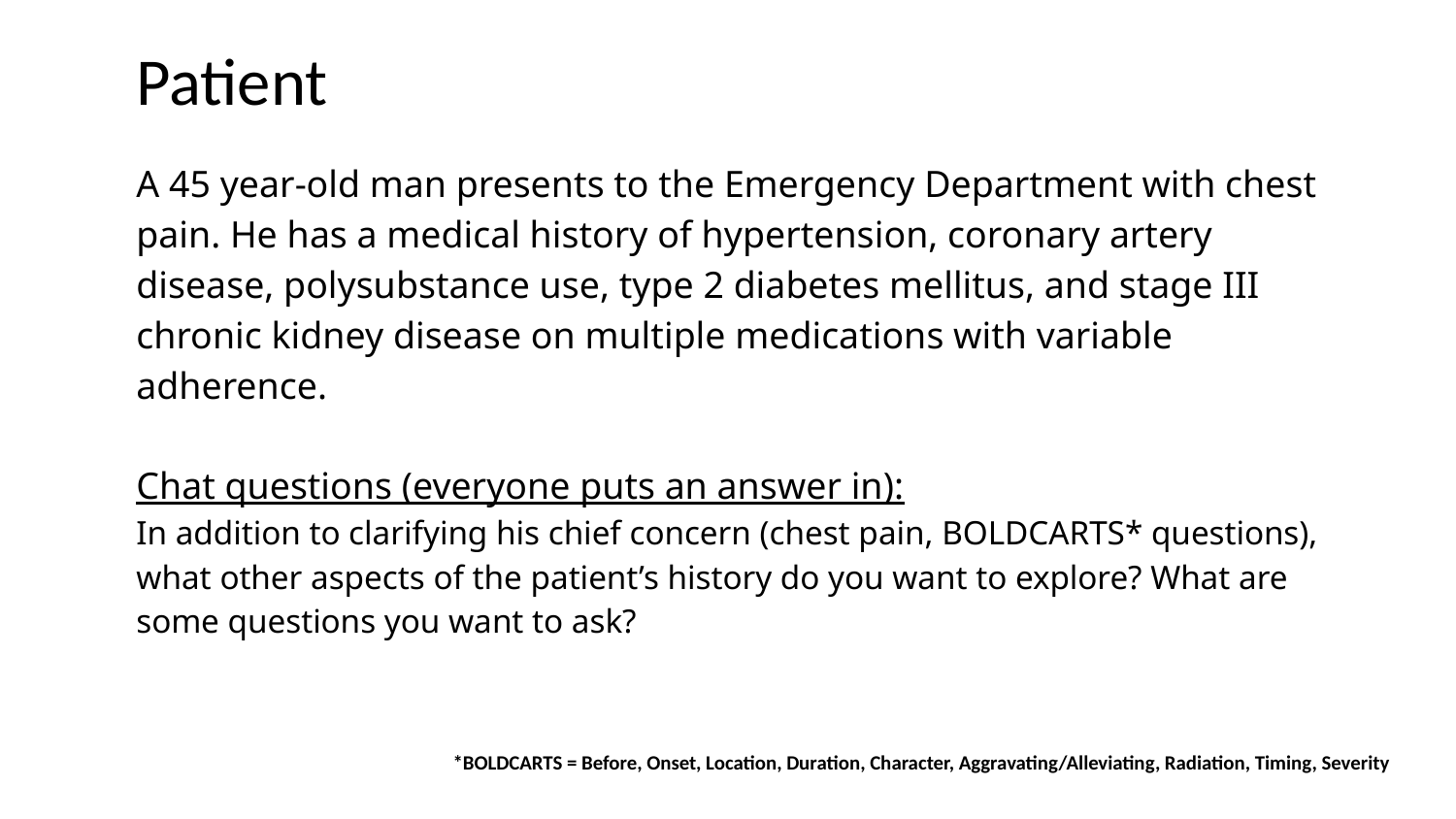

# Patient
A 45 year-old man presents to the Emergency Department with chest pain. He has a medical history of hypertension, coronary artery disease, polysubstance use, type 2 diabetes mellitus, and stage III chronic kidney disease on multiple medications with variable adherence.
Chat questions (everyone puts an answer in):
In addition to clarifying his chief concern (chest pain, BOLDCARTS* questions), what other aspects of the patient’s history do you want to explore? What are some questions you want to ask?
*BOLDCARTS = Before, Onset, Location, Duration, Character, Aggravating/Alleviating, Radiation, Timing, Severity

## Slide 7
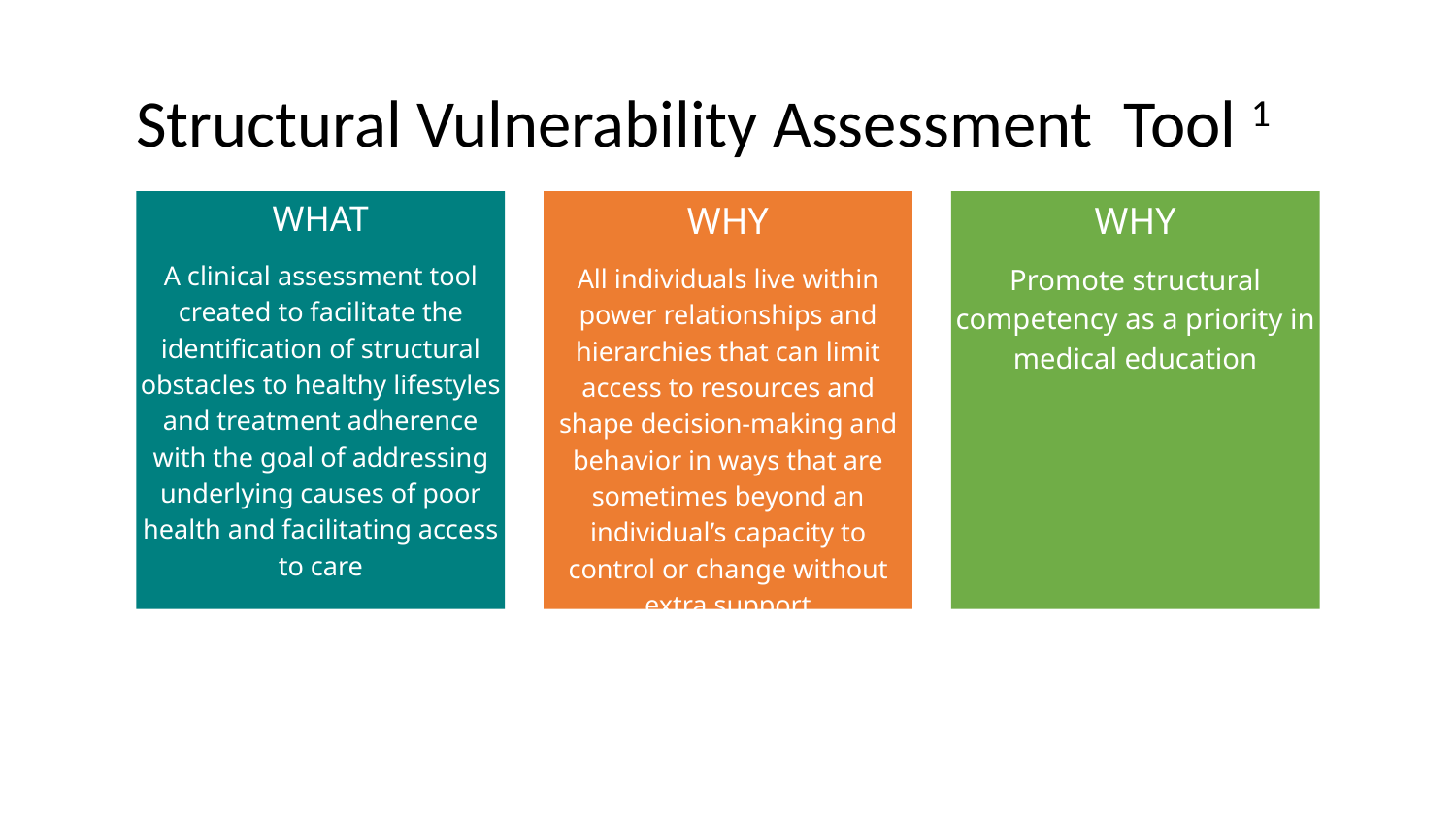

# Structural Vulnerability Assessment  Tool 1
WHAT
A clinical assessment tool created to facilitate the identification of structural obstacles to healthy lifestyles and treatment adherence with the goal of addressing underlying causes of poor health and facilitating access to care
WHY
All individuals live within power relationships and hierarchies that can limit access to resources and shape decision-making and behavior in ways that are sometimes beyond an individual’s capacity to control or change without extra support
WHY
Promote structural competency as a priority in medical education

## Slide 8
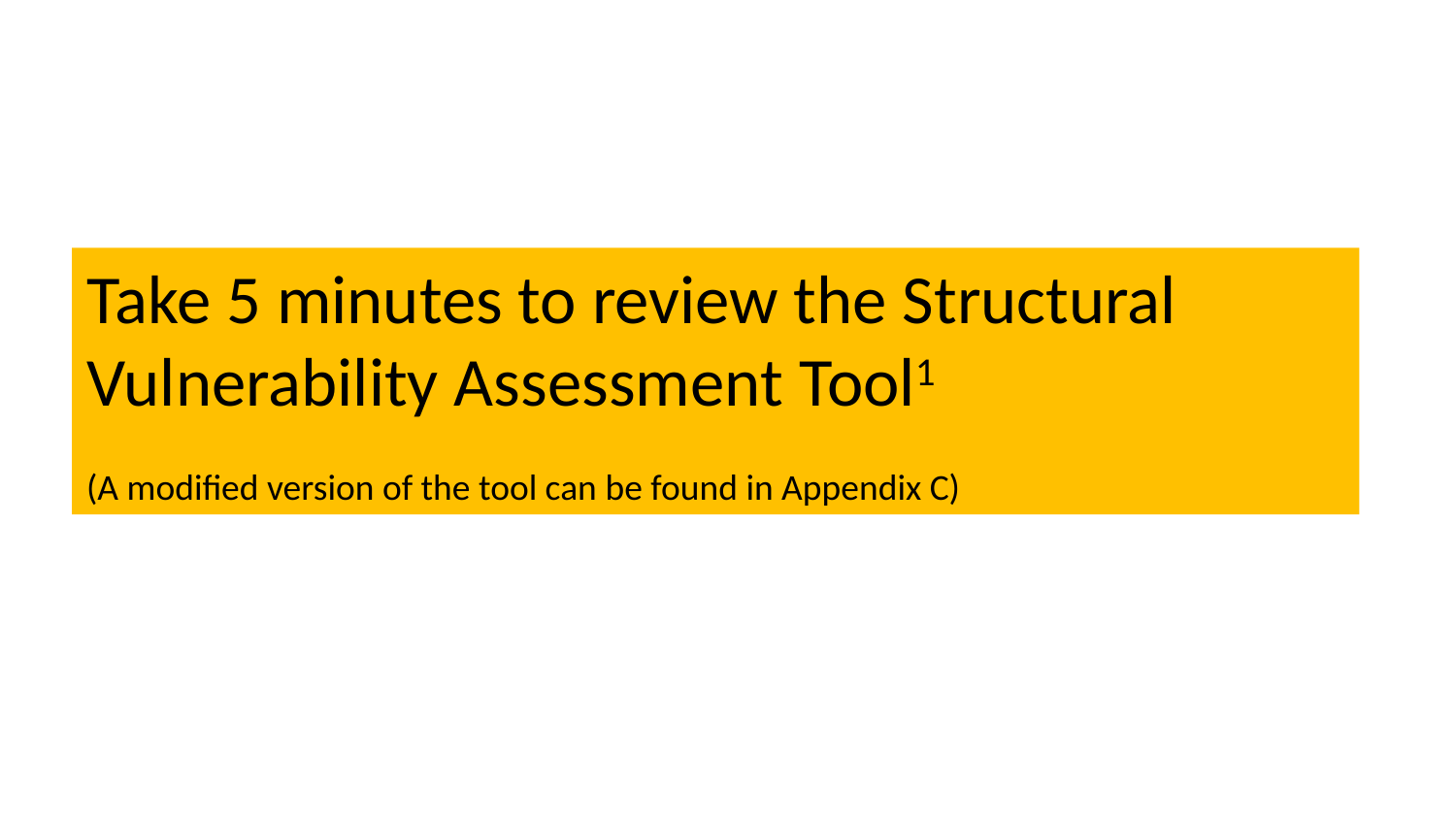

Take 5 minutes to review the Structural Vulnerability Assessment Tool1
(A modified version of the tool can be found in Appendix C)

## Slide 9
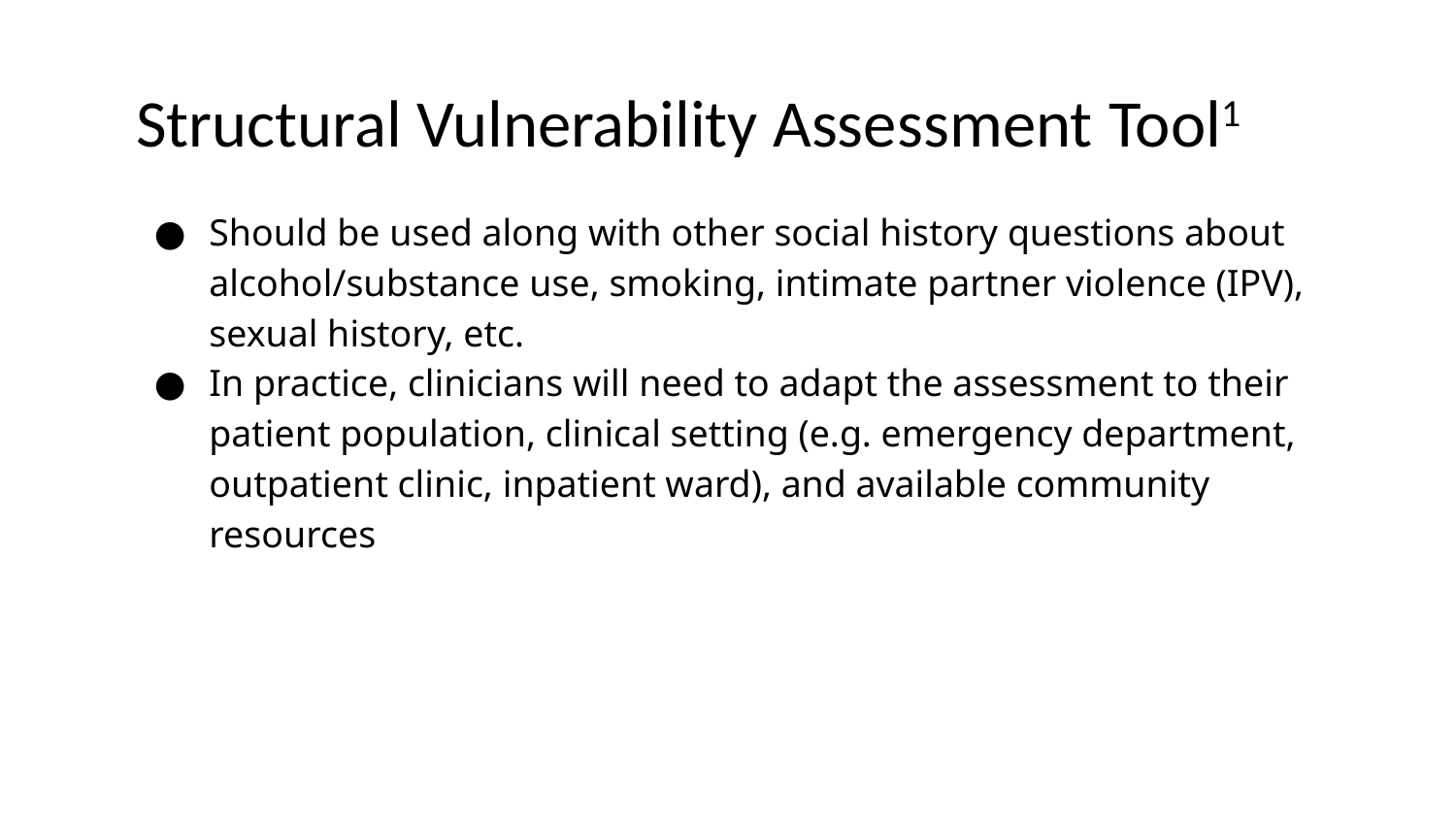

# Structural Vulnerability Assessment Tool1
Should be used along with other social history questions about alcohol/substance use, smoking, intimate partner violence (IPV), sexual history, etc.
In practice, clinicians will need to adapt the assessment to their patient population, clinical setting (e.g. emergency department, outpatient clinic, inpatient ward), and available community resources

## Slide 10
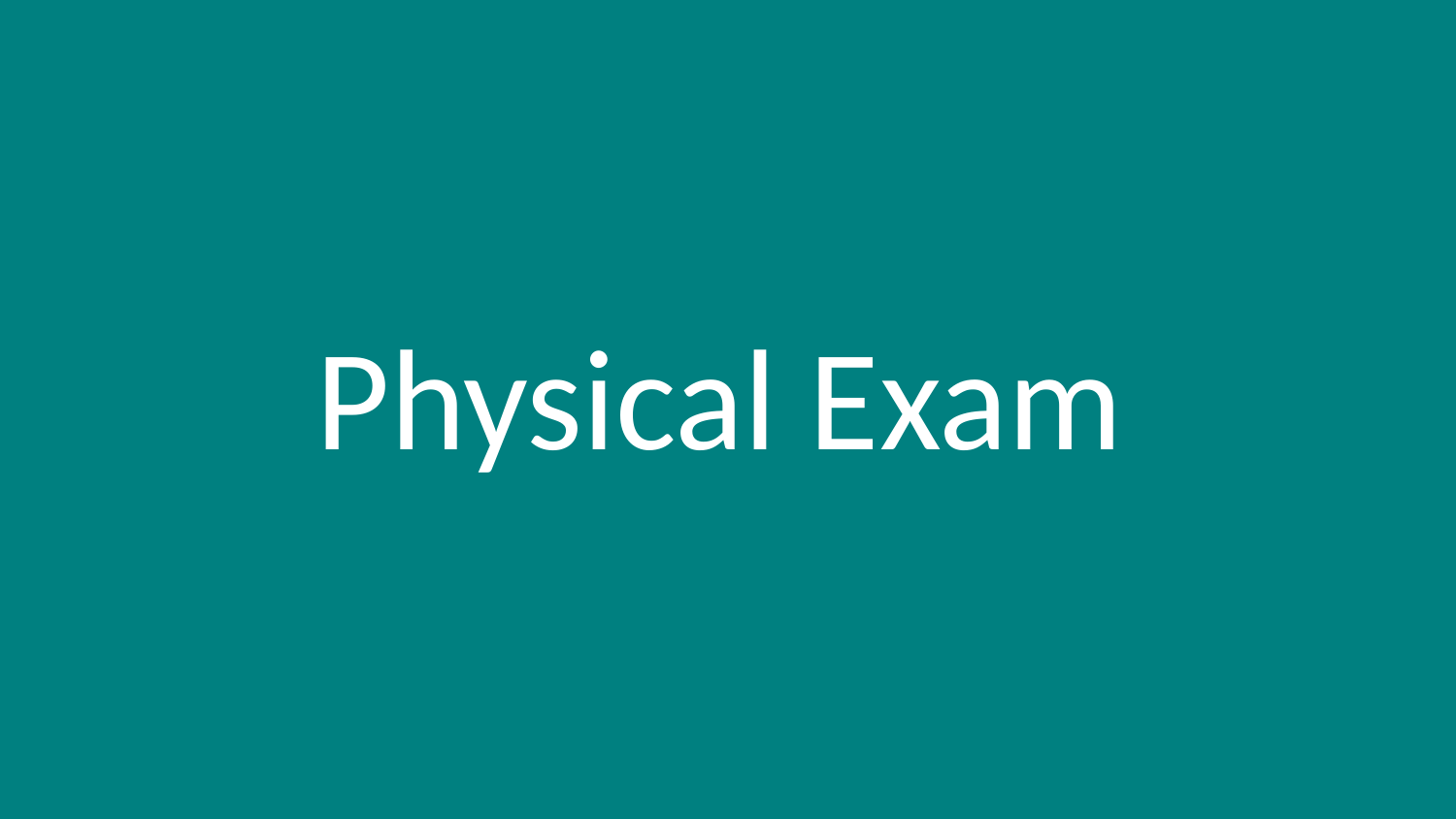

# Physical Exam

## Slide 11
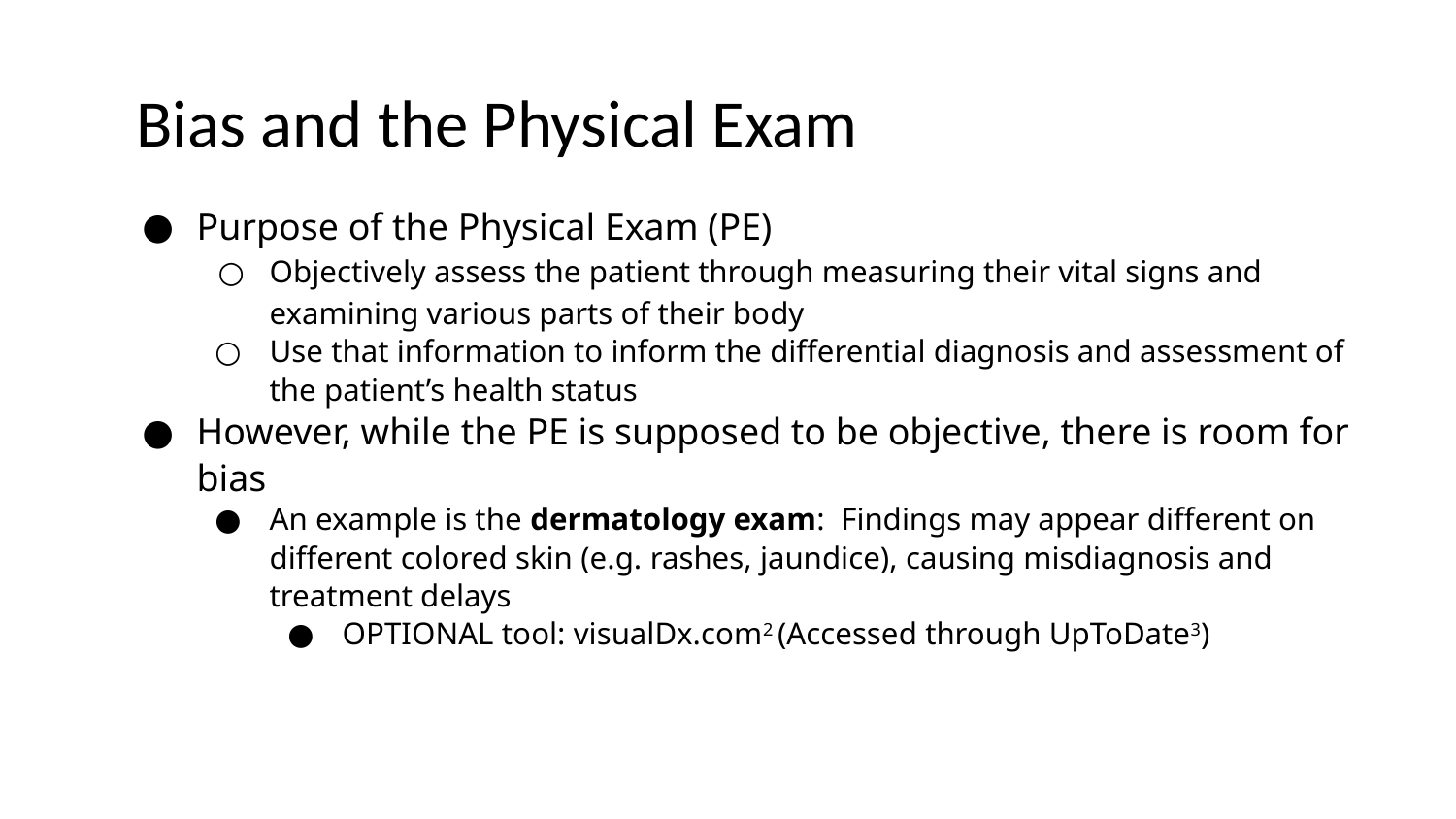

# Bias and the Physical Exam
Purpose of the Physical Exam (PE)
Objectively assess the patient through measuring their vital signs and examining various parts of their body
Use that information to inform the differential diagnosis and assessment of the patient’s health status
However, while the PE is supposed to be objective, there is room for bias
An example is the dermatology exam:  Findings may appear different on different colored skin (e.g. rashes, jaundice), causing misdiagnosis and treatment delays
OPTIONAL tool: visualDx.com2 (Accessed through UpToDate3)

## Slide 12
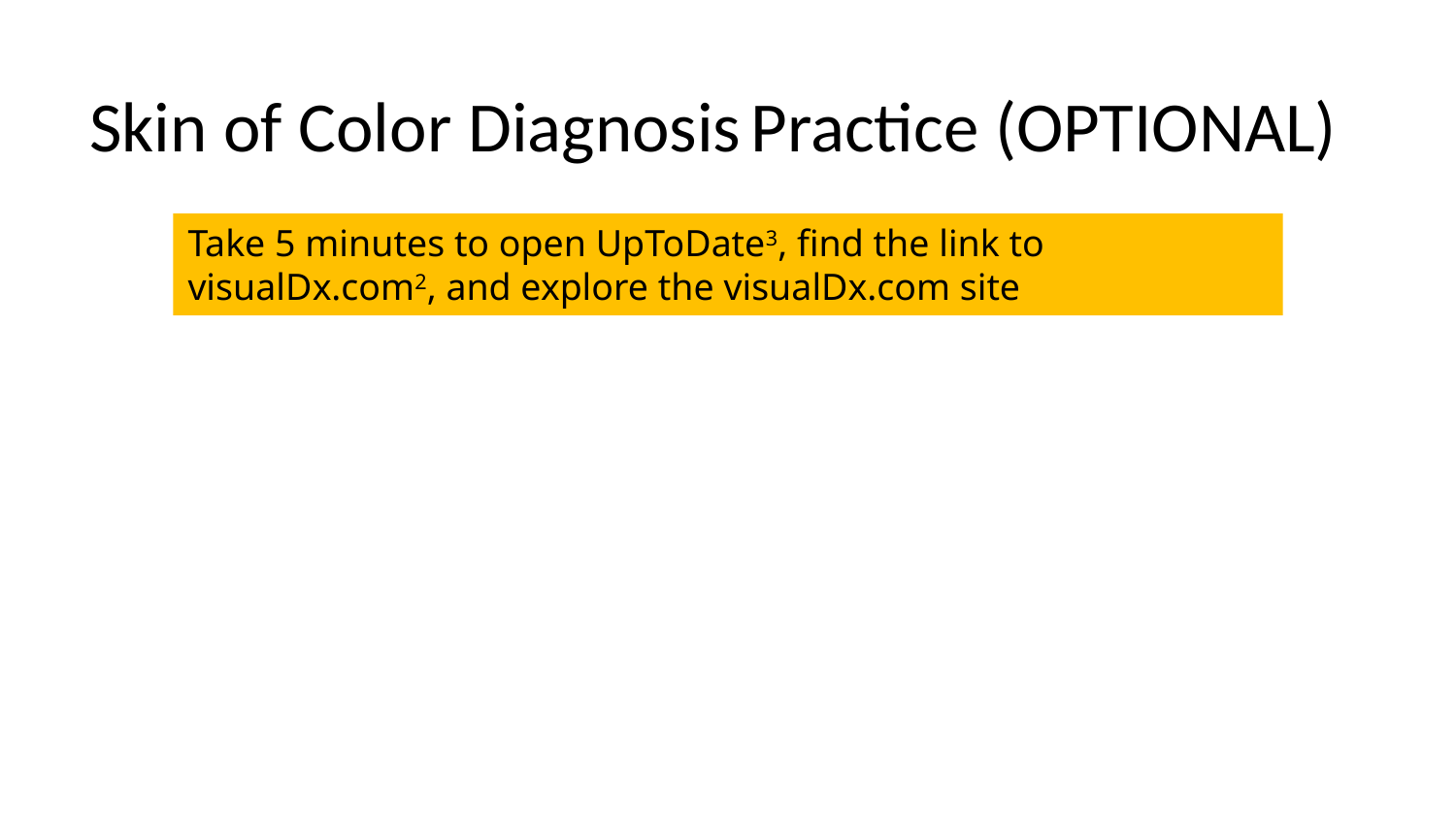

# Skin of Color Diagnosis Practice (OPTIONAL)
Take 5 minutes to open UpToDate3, find the link to visualDx.com2, and explore the visualDx.com site

## Slide 13
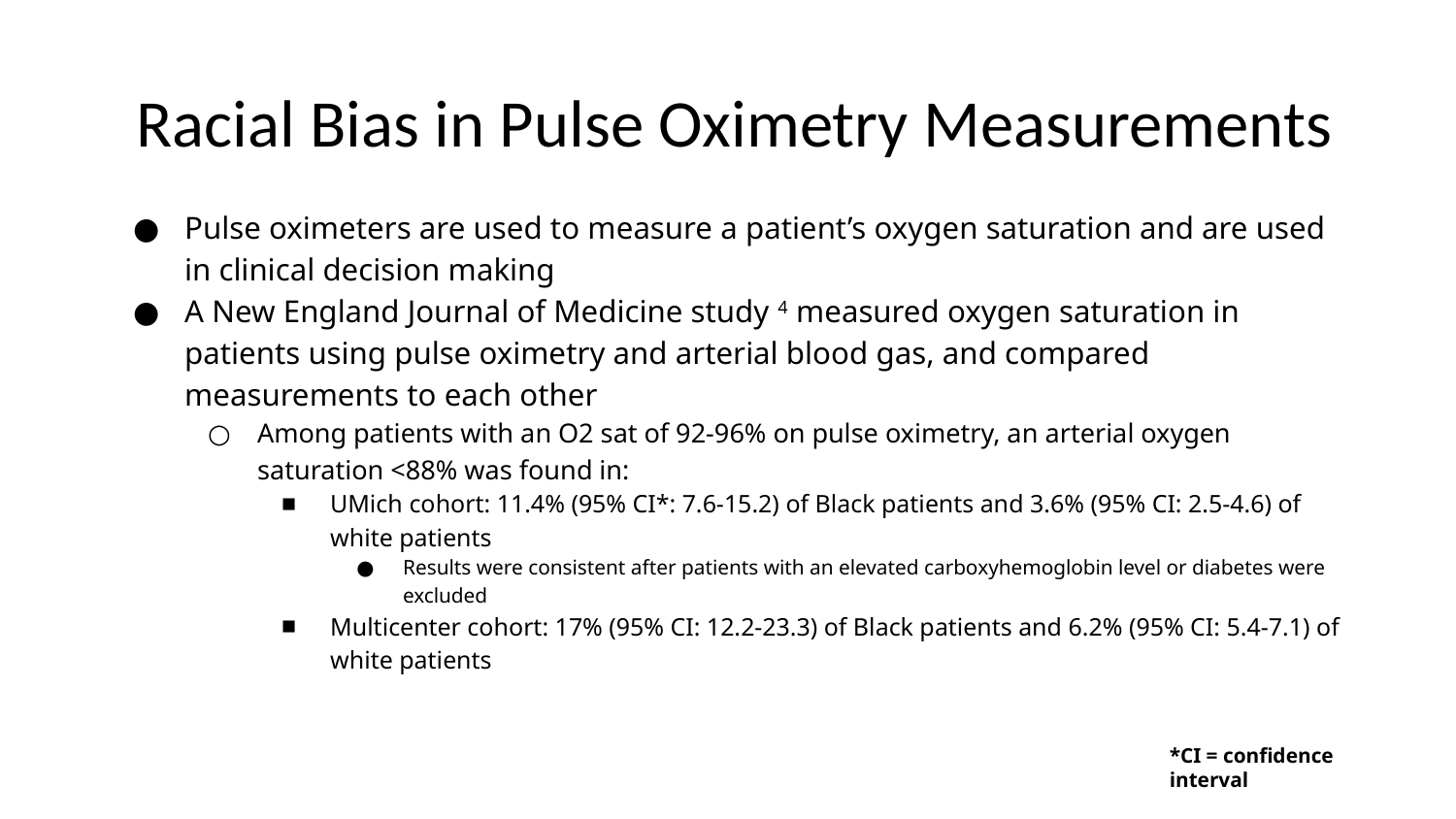

# Racial Bias in Pulse Oximetry Measurements
Pulse oximeters are used to measure a patient’s oxygen saturation and are used in clinical decision making
A New England Journal of Medicine study 4 measured oxygen saturation in patients using pulse oximetry and arterial blood gas, and compared measurements to each other
Among patients with an O2 sat of 92-96% on pulse oximetry, an arterial oxygen saturation <88% was found in:
UMich cohort: 11.4% (95% CI*: 7.6-15.2) of Black patients and 3.6% (95% CI: 2.5-4.6) of white patients
Results were consistent after patients with an elevated carboxyhemoglobin level or diabetes were excluded
Multicenter cohort: 17% (95% CI: 12.2-23.3) of Black patients and 6.2% (95% CI: 5.4-7.1) of white patients
*CI = confidence interval

## Slide 14
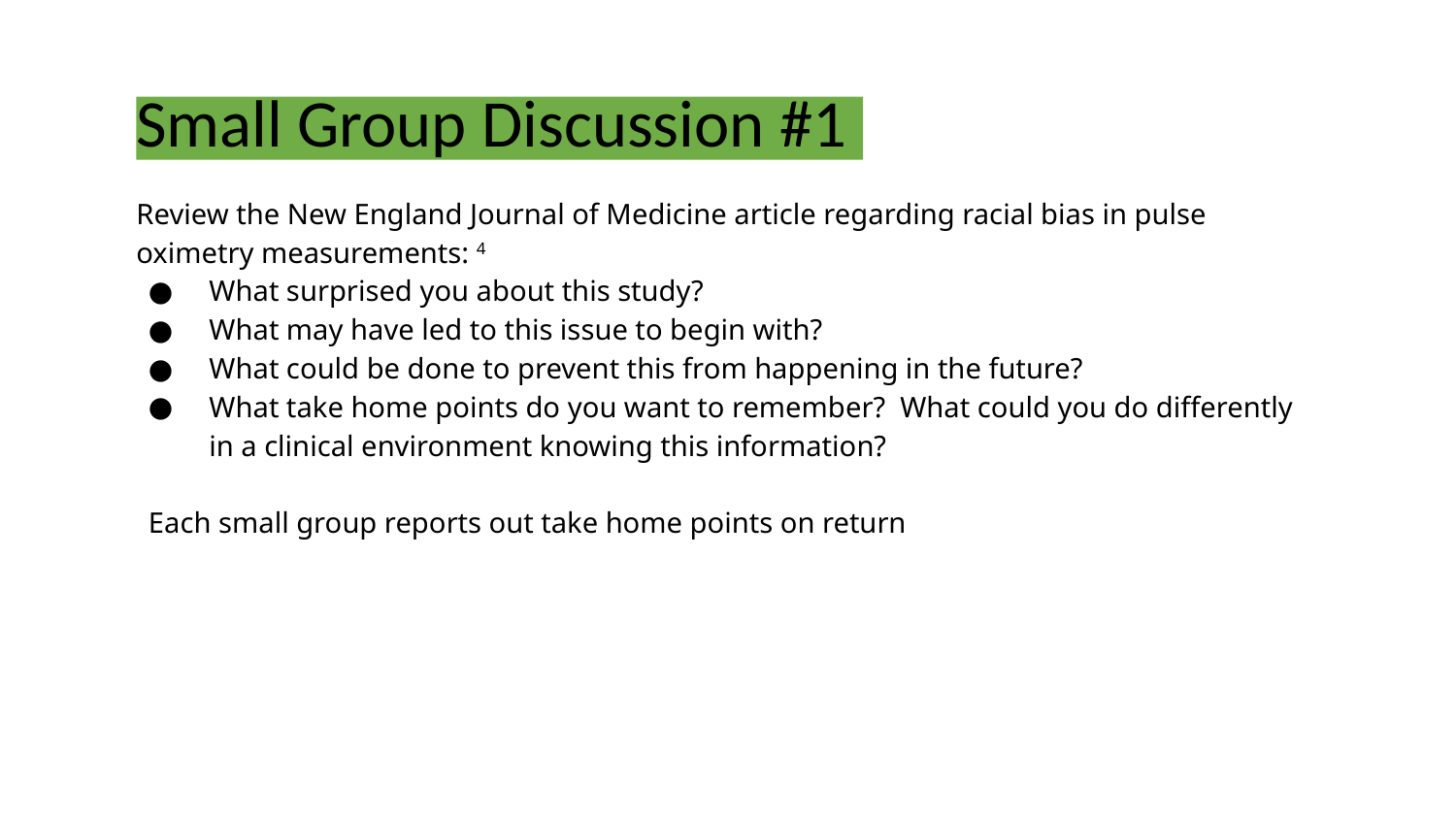

# Small Group Discussion #1
Review the New England Journal of Medicine article regarding racial bias in pulse oximetry measurements: 4
What surprised you about this study?
What may have led to this issue to begin with?
What could be done to prevent this from happening in the future?
What take home points do you want to remember?  What could you do differently in a clinical environment knowing this information?
Each small group reports out take home points on return

## Slide 15
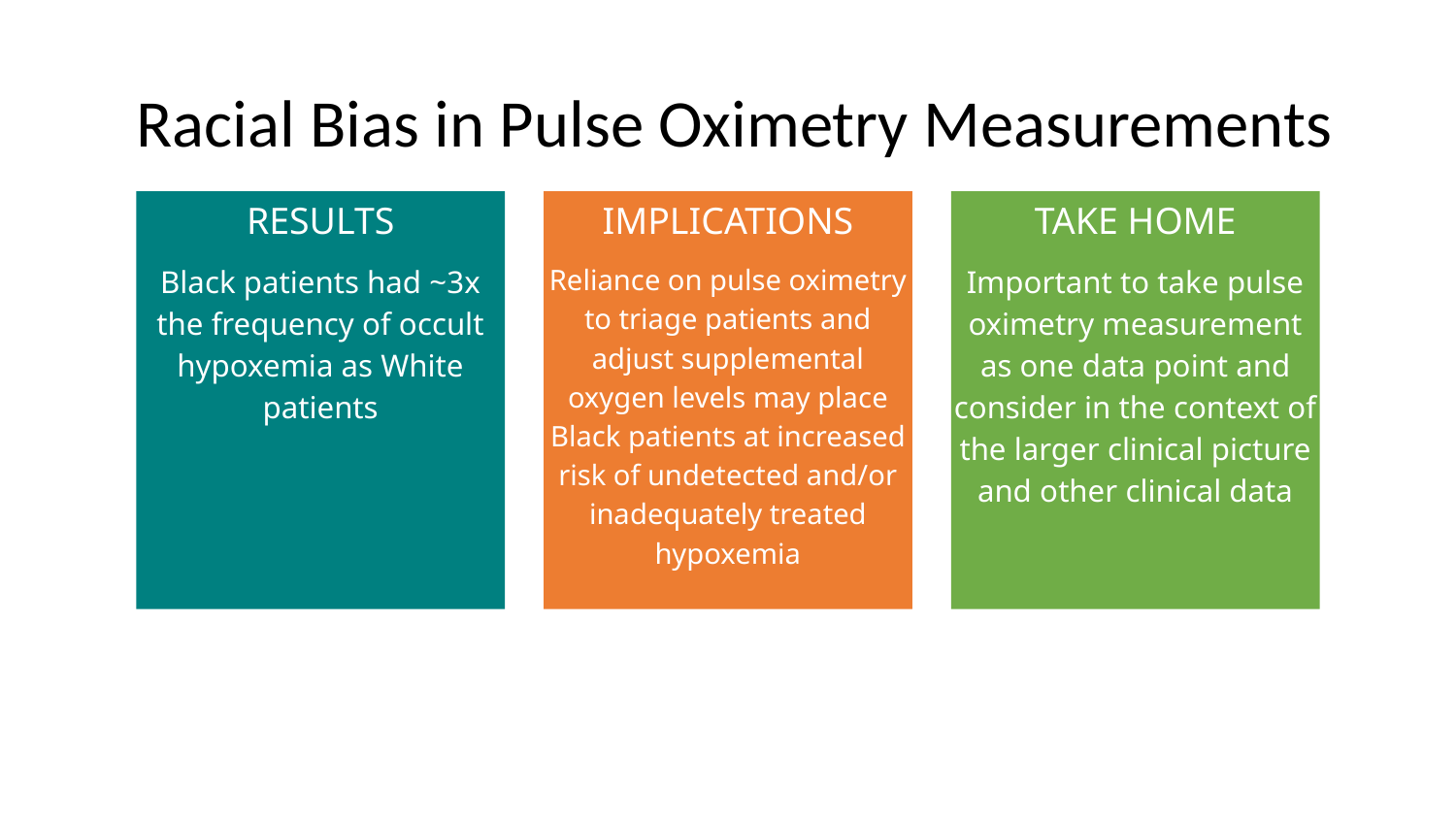

# Racial Bias in Pulse Oximetry Measurements
RESULTS
Black patients had ~3x the frequency of occult hypoxemia as White patients
IMPLICATIONS
Reliance on pulse oximetry to triage patients and adjust supplemental oxygen levels may place Black patients at increased risk of undetected and/or inadequately treated hypoxemia
TAKE HOME
Important to take pulse oximetry measurement as one data point and consider in the context of the larger clinical picture and other clinical data

## Slide 16
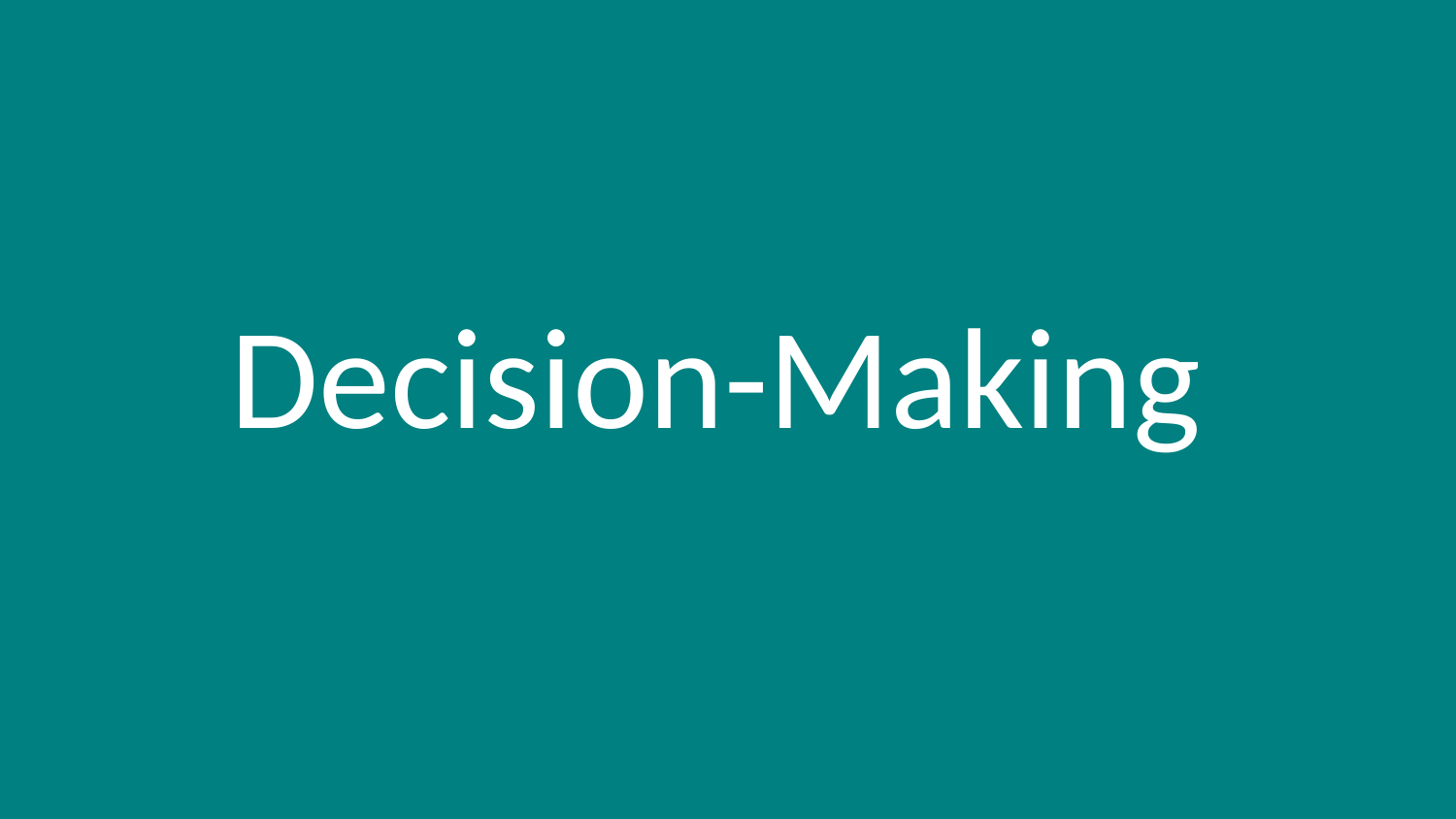

# Decision-Making

## Slide 17
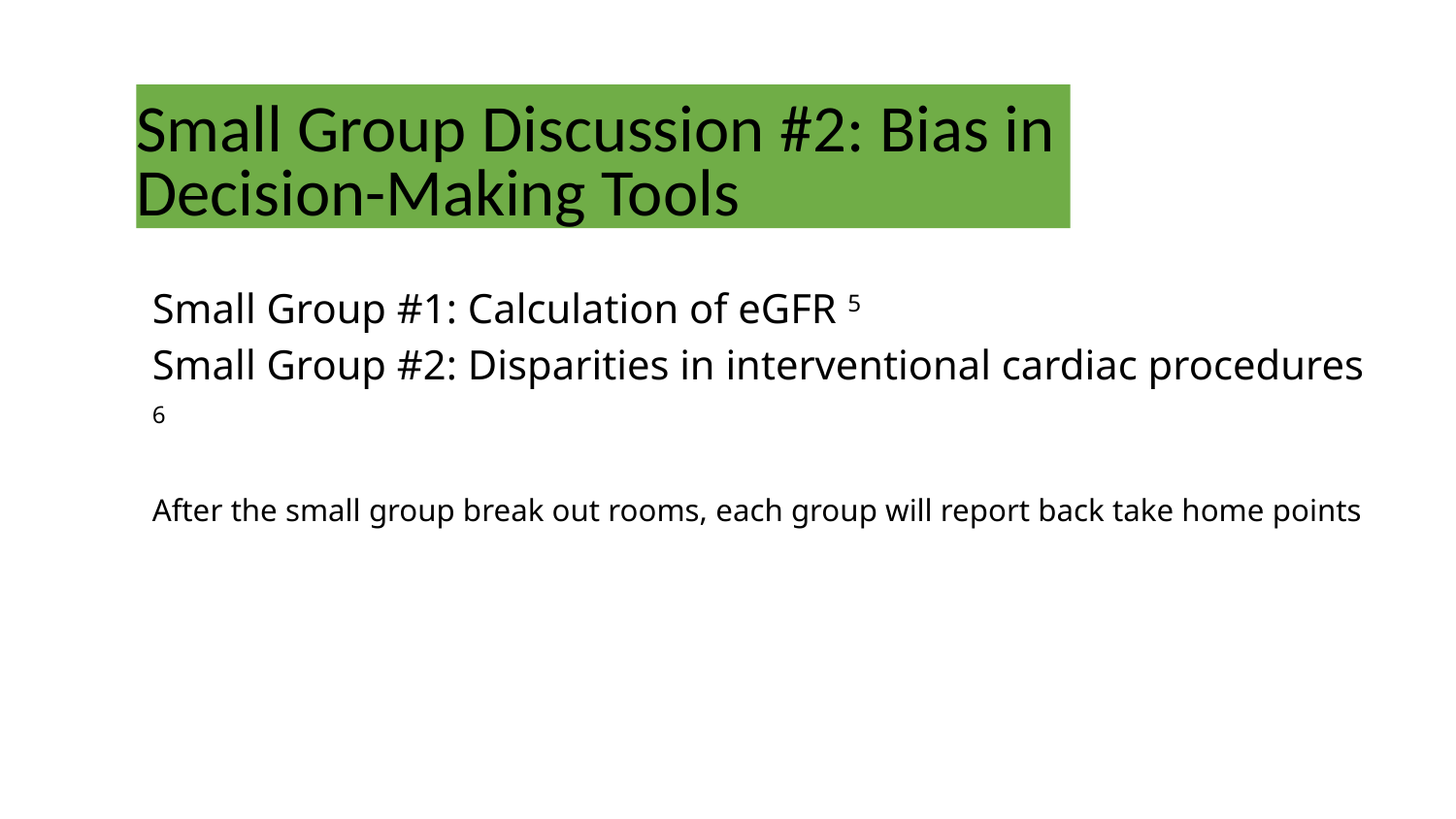

# Small Group Discussion #2: Bias in Decision-Making Tools
Small Group #1: Calculation of eGFR 5
Small Group #2: Disparities in interventional cardiac procedures 6
After the small group break out rooms, each group will report back take home points

## Slide 18
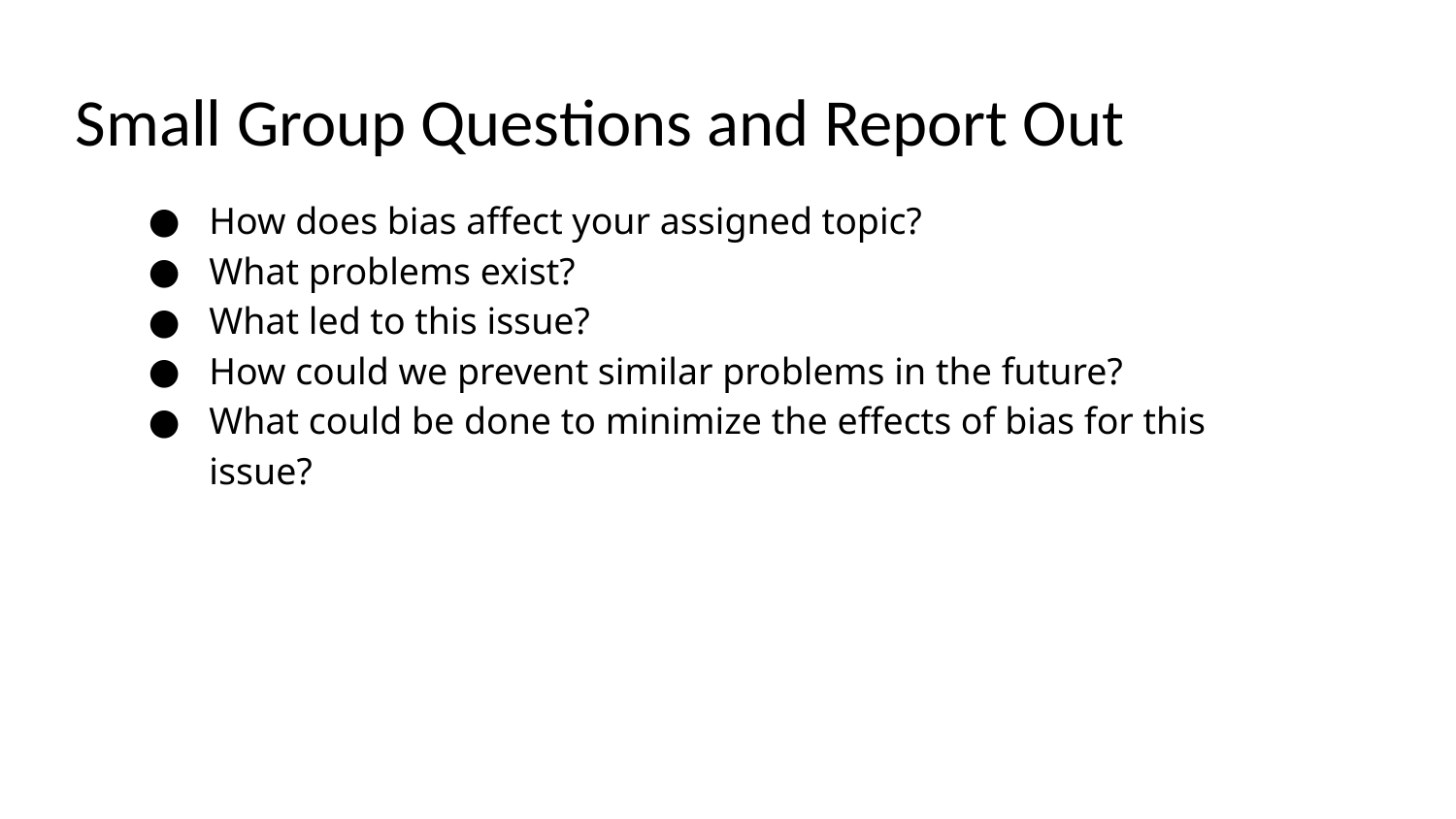

# Small Group Questions and Report Out
How does bias affect your assigned topic?
What problems exist?
What led to this issue?
How could we prevent similar problems in the future?
What could be done to minimize the effects of bias for this issue?

## Slide 19
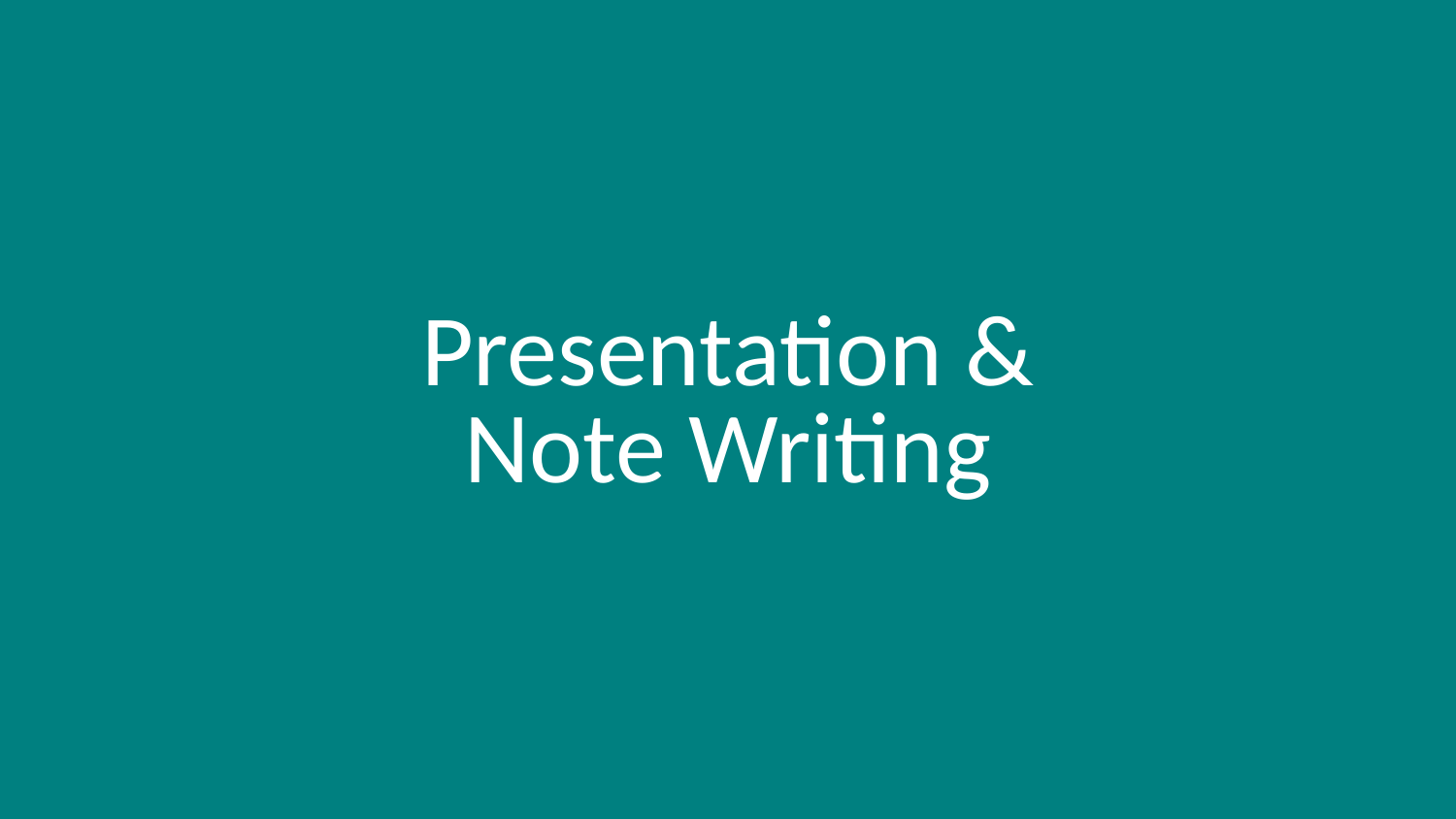

# Presentation & Note Writing

## Slide 20
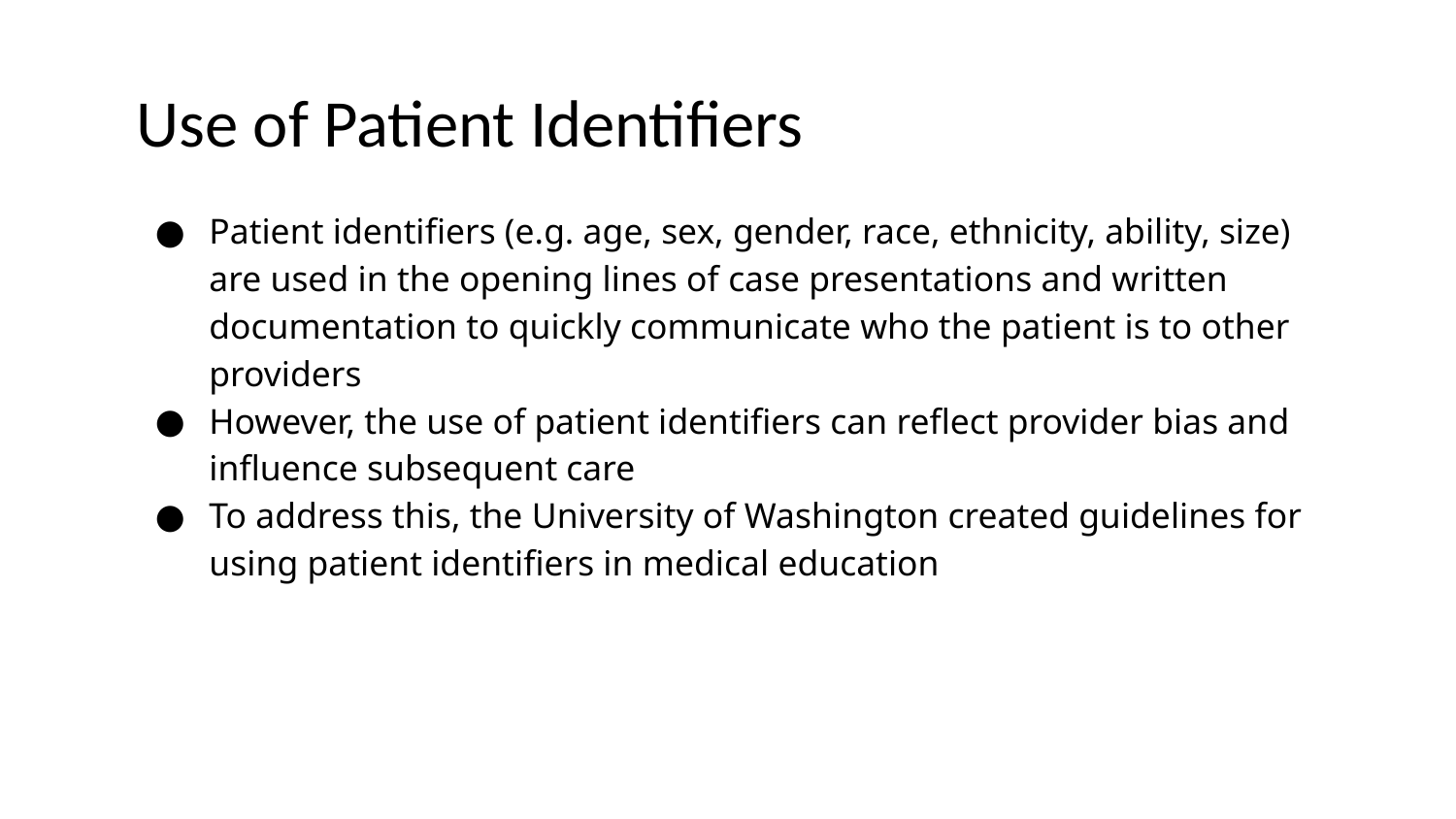

# Use of Patient Identifiers
Patient identifiers (e.g. age, sex, gender, race, ethnicity, ability, size) are used in the opening lines of case presentations and written documentation to quickly communicate who the patient is to other providers
However, the use of patient identifiers can reflect provider bias and influence subsequent care
To address this, the University of Washington created guidelines for using patient identifiers in medical education

## Slide 21
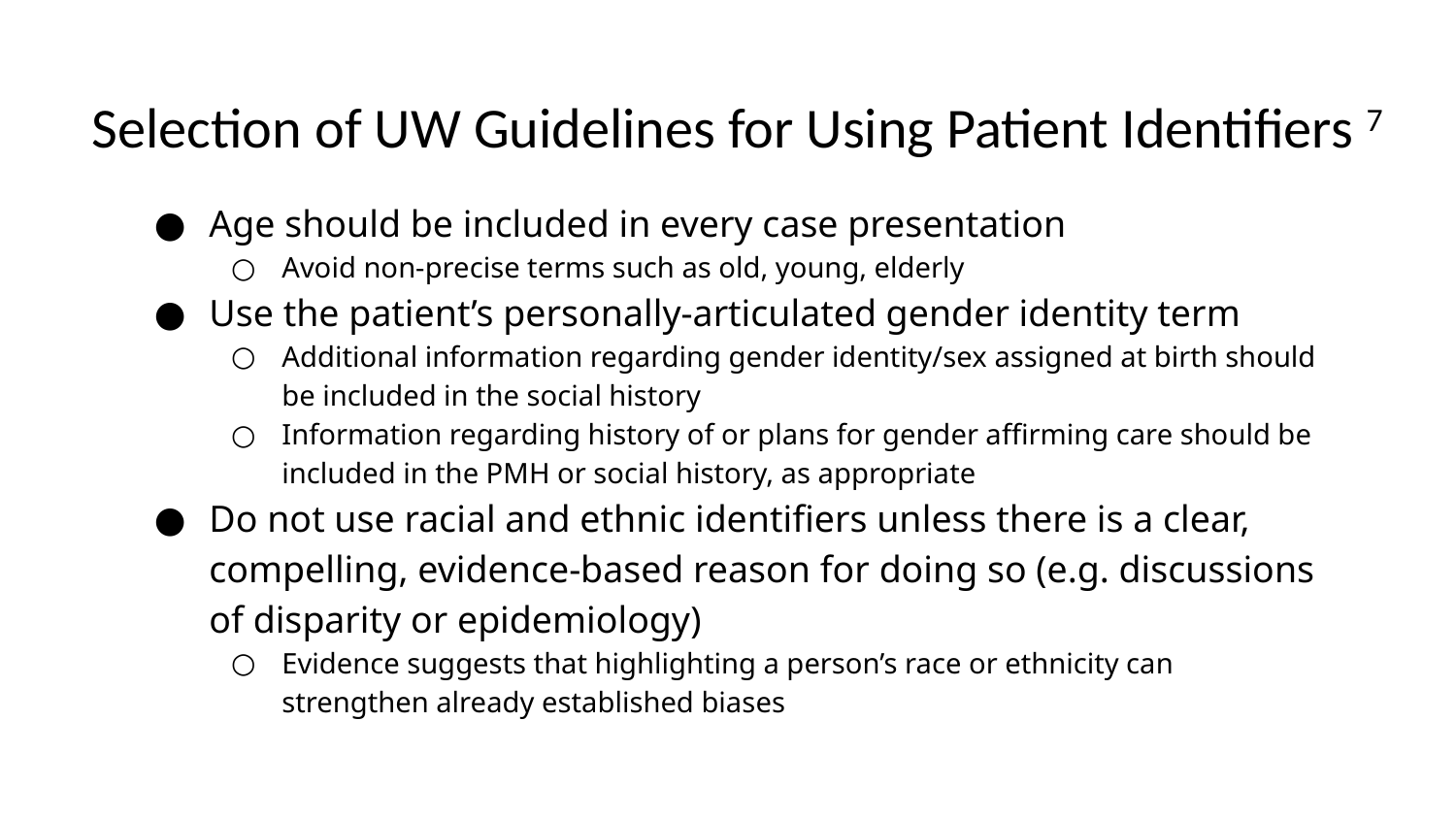

# Selection of UW Guidelines for Using Patient Identifiers 7
Age should be included in every case presentation
Avoid non-precise terms such as old, young, elderly
Use the patient’s personally-articulated gender identity term
Additional information regarding gender identity/sex assigned at birth should be included in the social history
Information regarding history of or plans for gender affirming care should be included in the PMH or social history, as appropriate
Do not use racial and ethnic identifiers unless there is a clear, compelling, evidence-based reason for doing so (e.g. discussions of disparity or epidemiology)
Evidence suggests that highlighting a person’s race or ethnicity can strengthen already established biases

## Slide 22
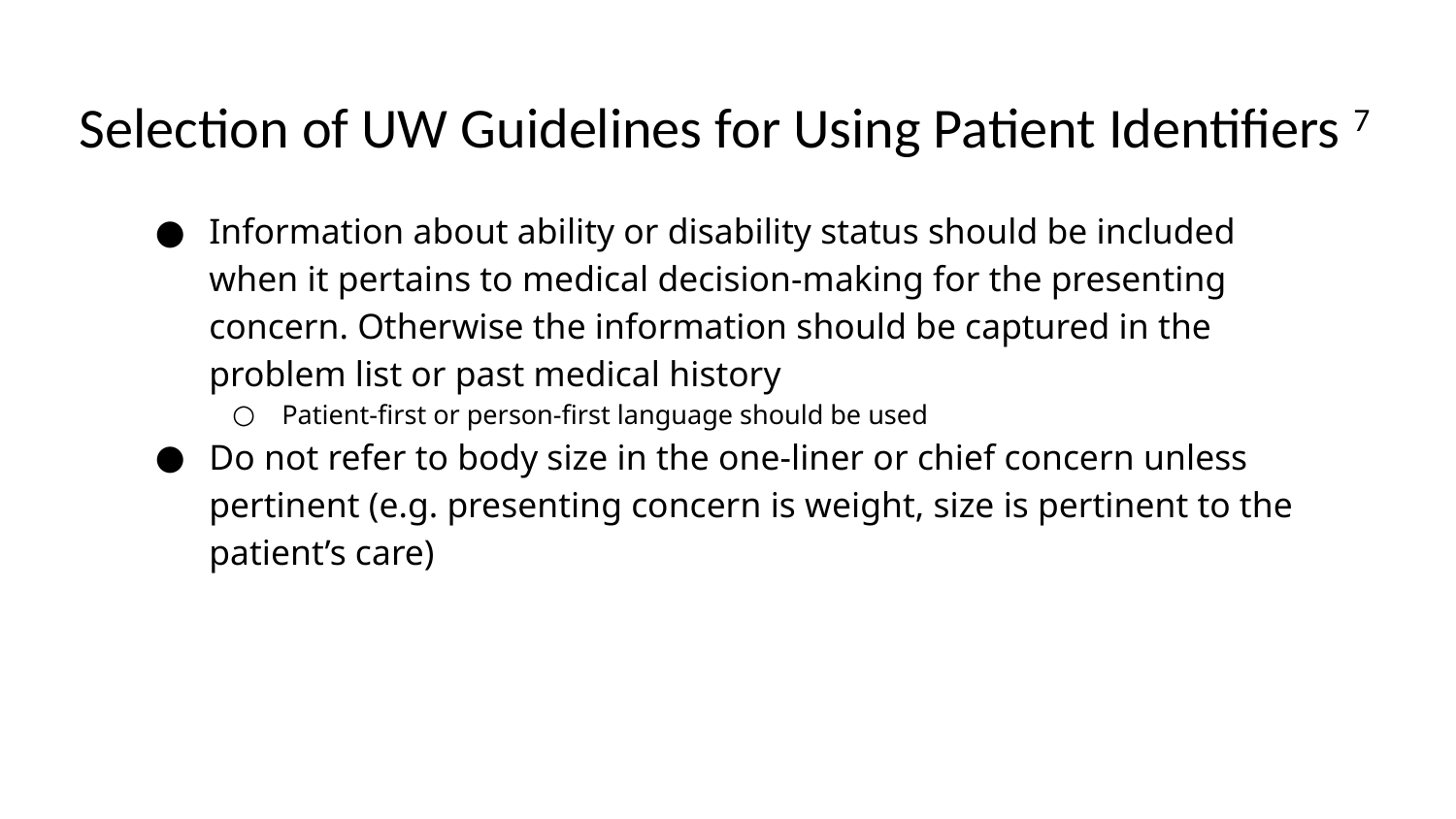

# Selection of UW Guidelines for Using Patient Identifiers 7
Information about ability or disability status should be included when it pertains to medical decision-making for the presenting concern. Otherwise the information should be captured in the problem list or past medical history
Patient-first or person-first language should be used
Do not refer to body size in the one-liner or chief concern unless pertinent (e.g. presenting concern is weight, size is pertinent to the patient’s care)

## Slide 23
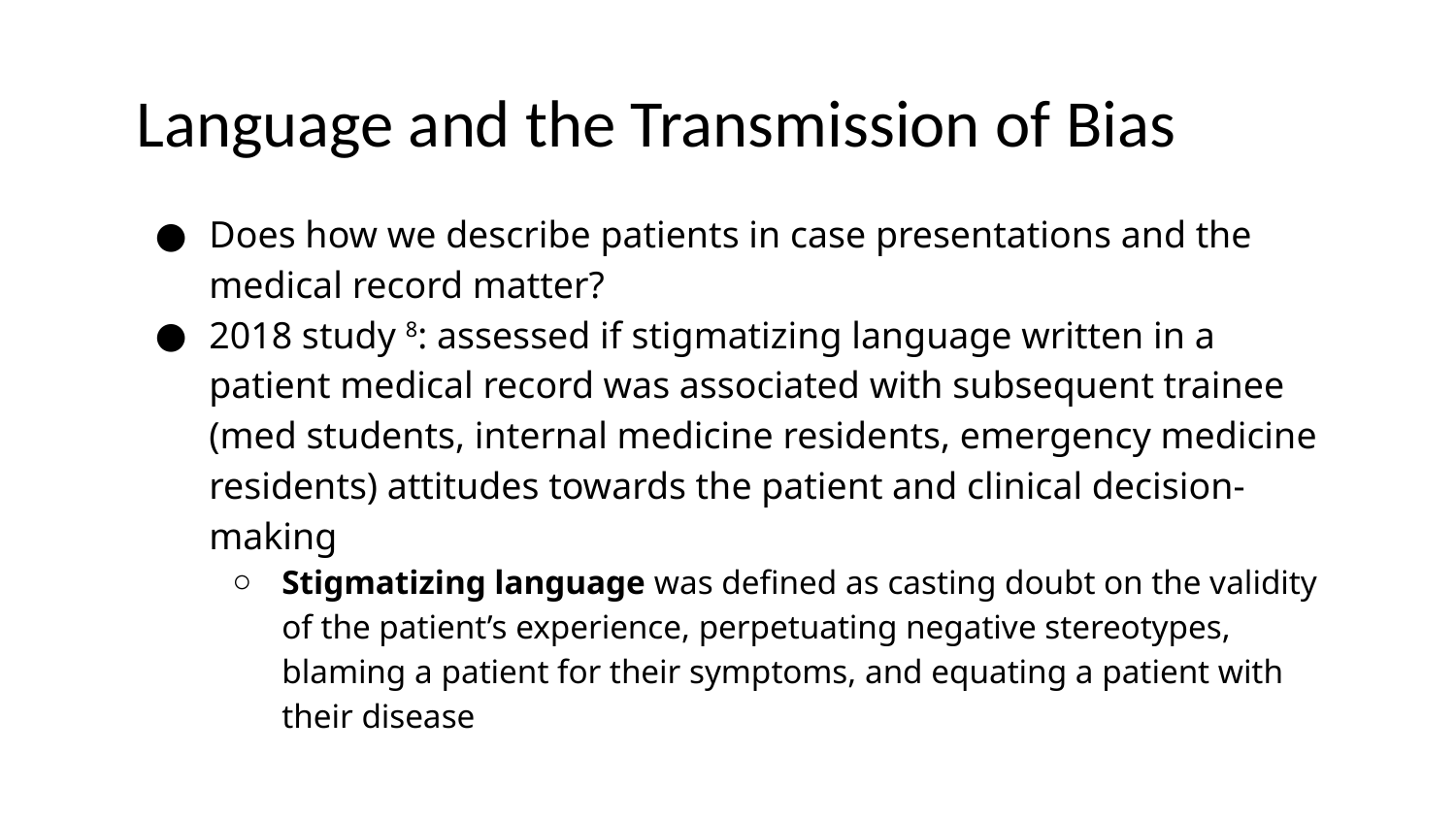

# Language and the Transmission of Bias
Does how we describe patients in case presentations and the medical record matter?
2018 study 8: assessed if stigmatizing language written in a patient medical record was associated with subsequent trainee (med students, internal medicine residents, emergency medicine residents) attitudes towards the patient and clinical decision-making
Stigmatizing language was defined as casting doubt on the validity of the patient’s experience, perpetuating negative stereotypes, blaming a patient for their symptoms, and equating a patient with their disease

## Slide 24
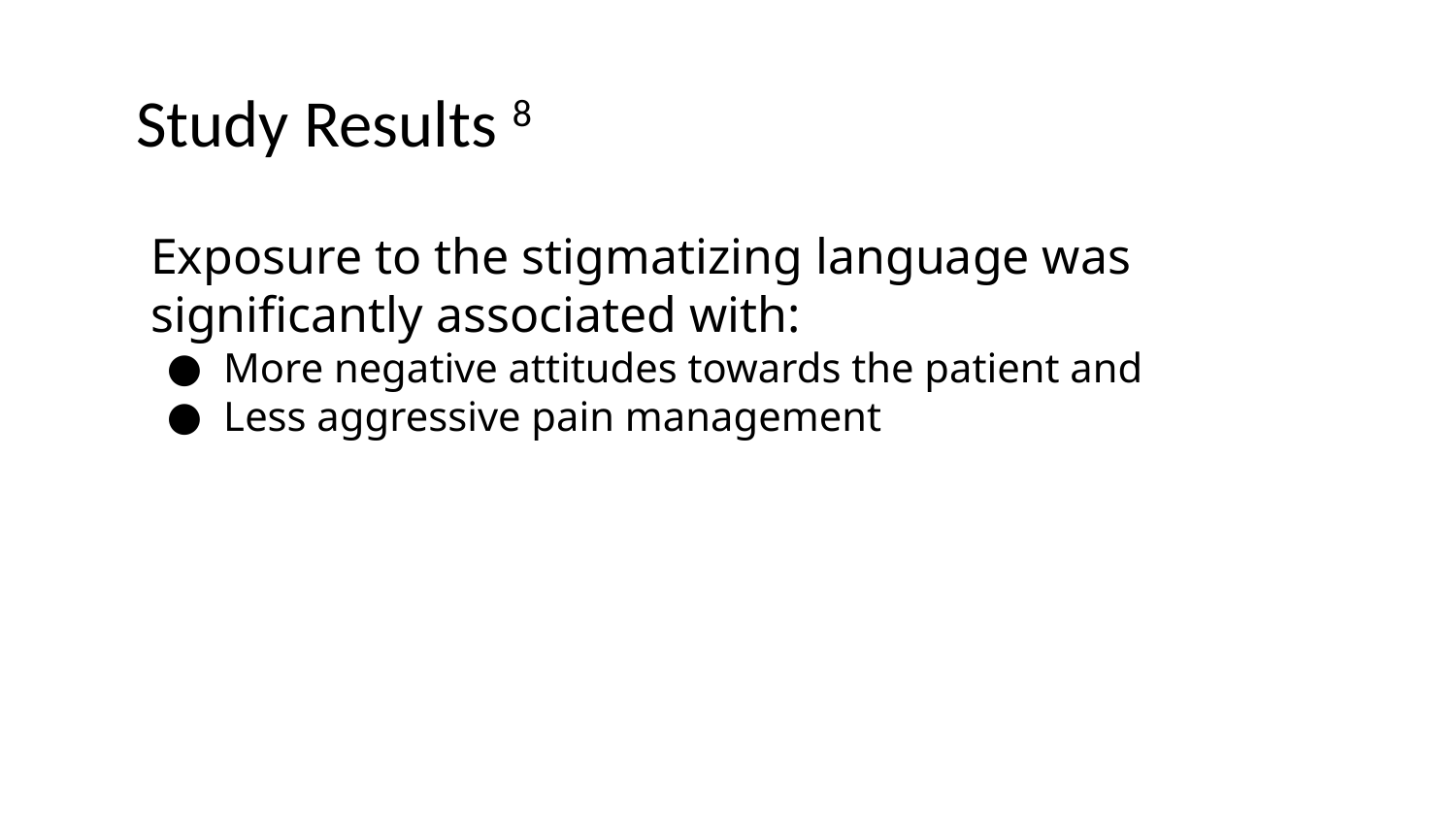

# Study Results 8
Exposure to the stigmatizing language was significantly associated with:
More negative attitudes towards the patient and
Less aggressive pain management

## Slide 25
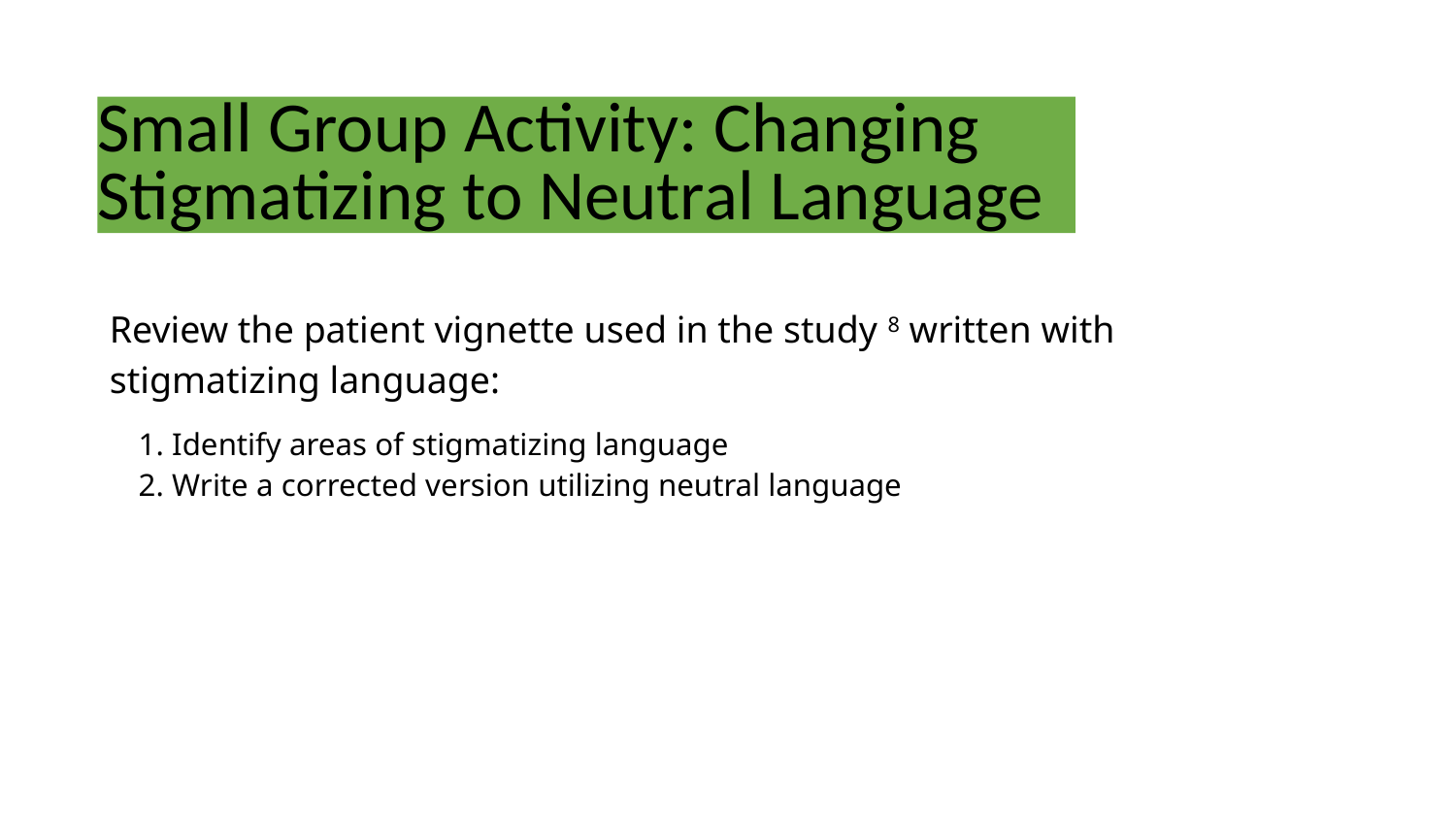

# Small Group Activity: Changing Stigmatizing to Neutral Language
Review the patient vignette used in the study 8 written with stigmatizing language:
1. Identify areas of stigmatizing language
2. Write a corrected version utilizing neutral language

## Slide 26
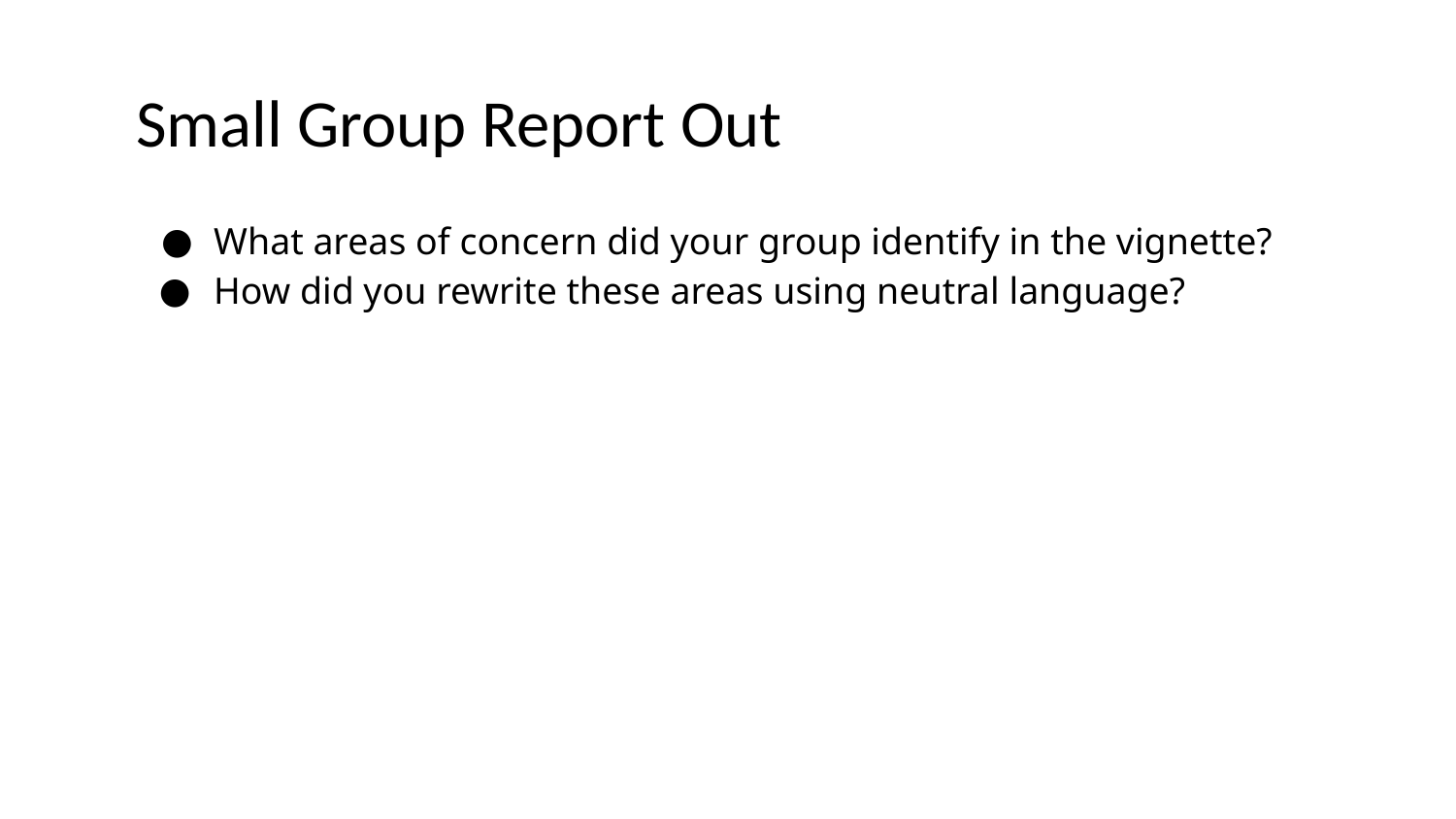

# Small Group Report Out
What areas of concern did your group identify in the vignette?
How did you rewrite these areas using neutral language?

## Slide 27
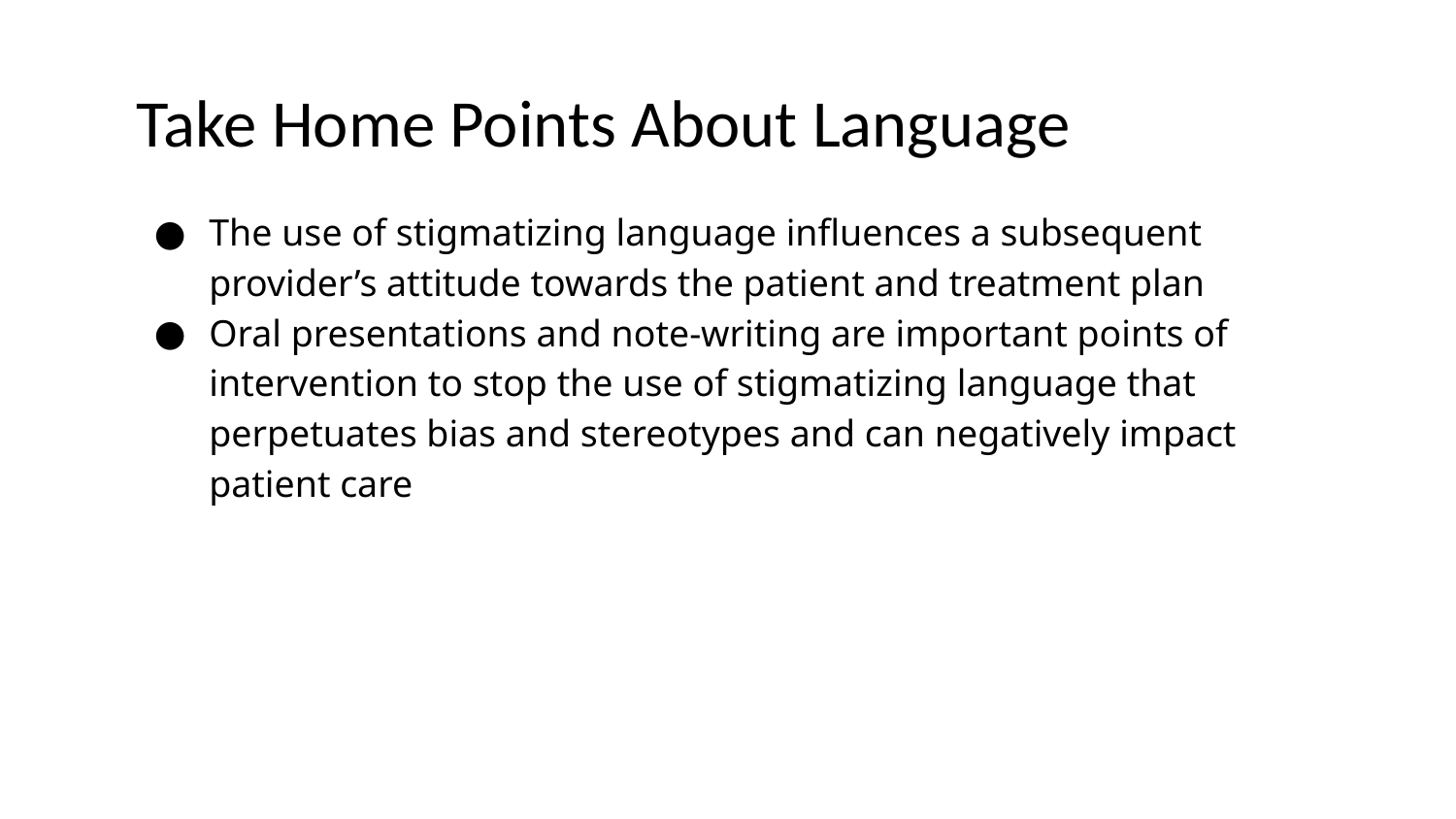

# Take Home Points About Language
The use of stigmatizing language influences a subsequent provider’s attitude towards the patient and treatment plan
Oral presentations and note-writing are important points of intervention to stop the use of stigmatizing language that perpetuates bias and stereotypes and can negatively impact patient care

## Slide 28
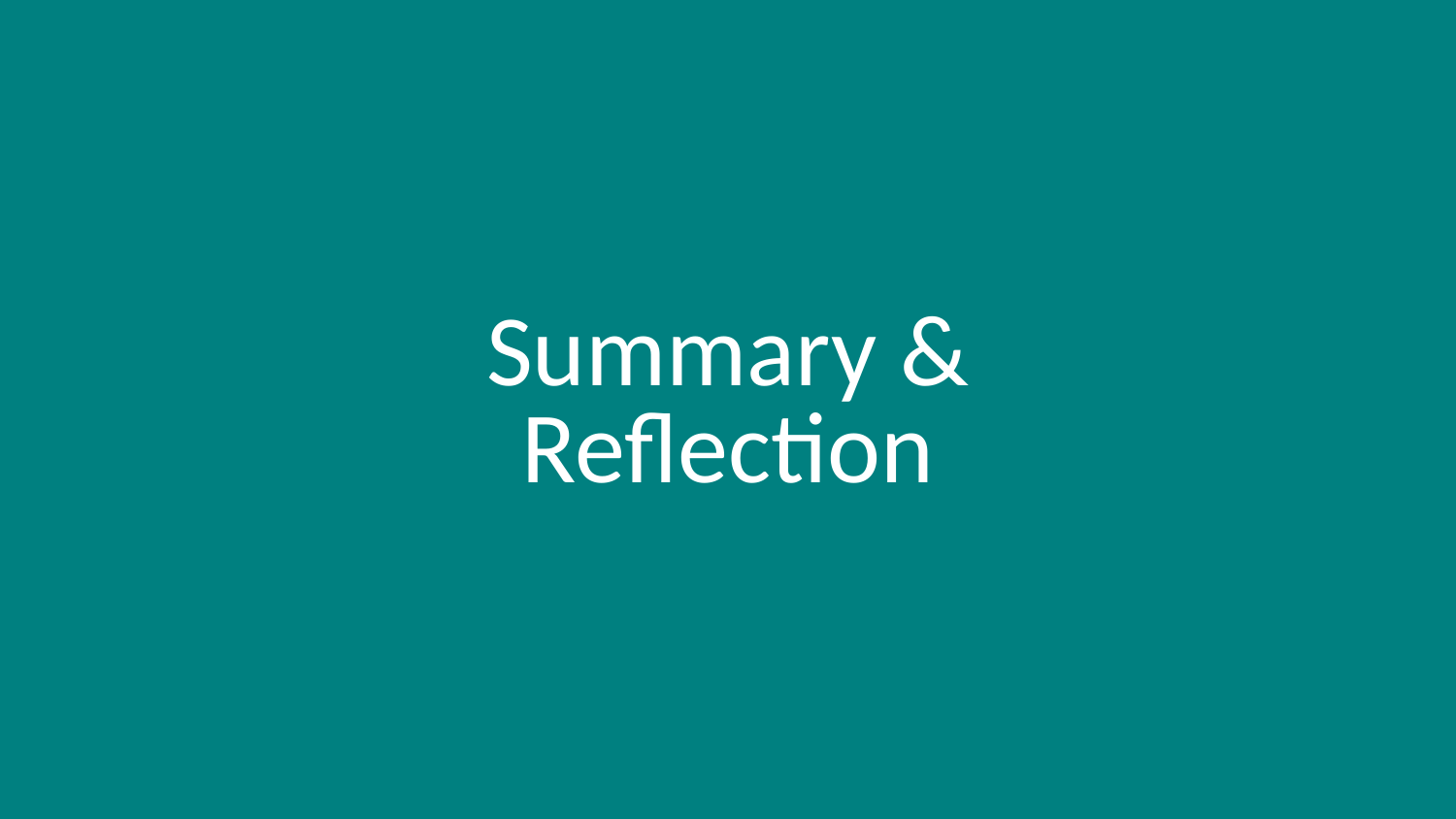

# Summary & Reflection

## Slide 29
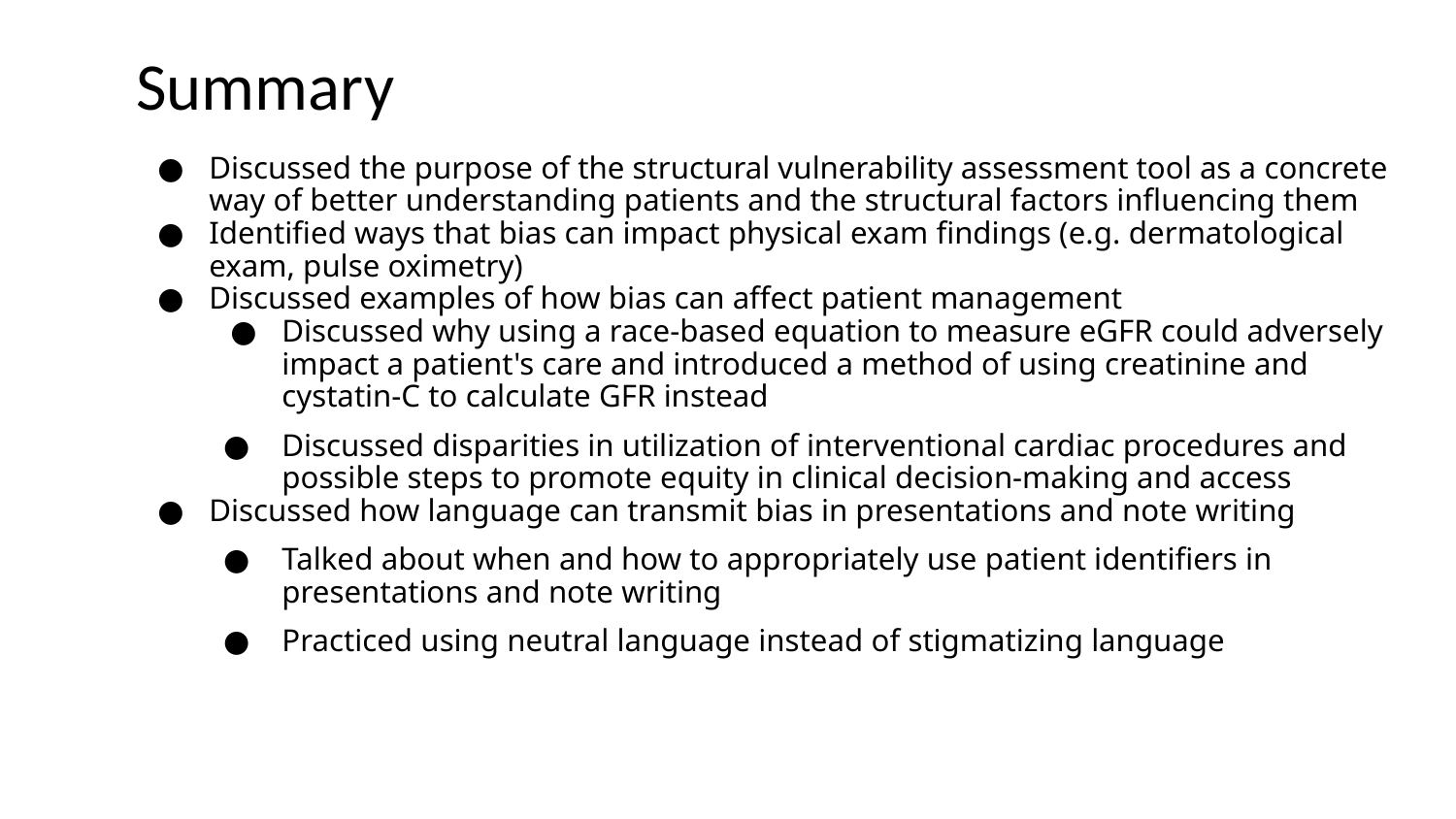

# Summary
Discussed the purpose of the structural vulnerability assessment tool as a concrete way of better understanding patients and the structural factors influencing them
Identified ways that bias can impact physical exam findings (e.g. dermatological exam, pulse oximetry)
Discussed examples of how bias can affect patient management
Discussed why using a race-based equation to measure eGFR could adversely impact a patient's care and introduced a method of using creatinine and cystatin-C to calculate GFR instead
Discussed disparities in utilization of interventional cardiac procedures and possible steps to promote equity in clinical decision-making and access
Discussed how language can transmit bias in presentations and note writing
Talked about when and how to appropriately use patient identifiers in presentations and note writing
Practiced using neutral language instead of stigmatizing language

## Slide 30
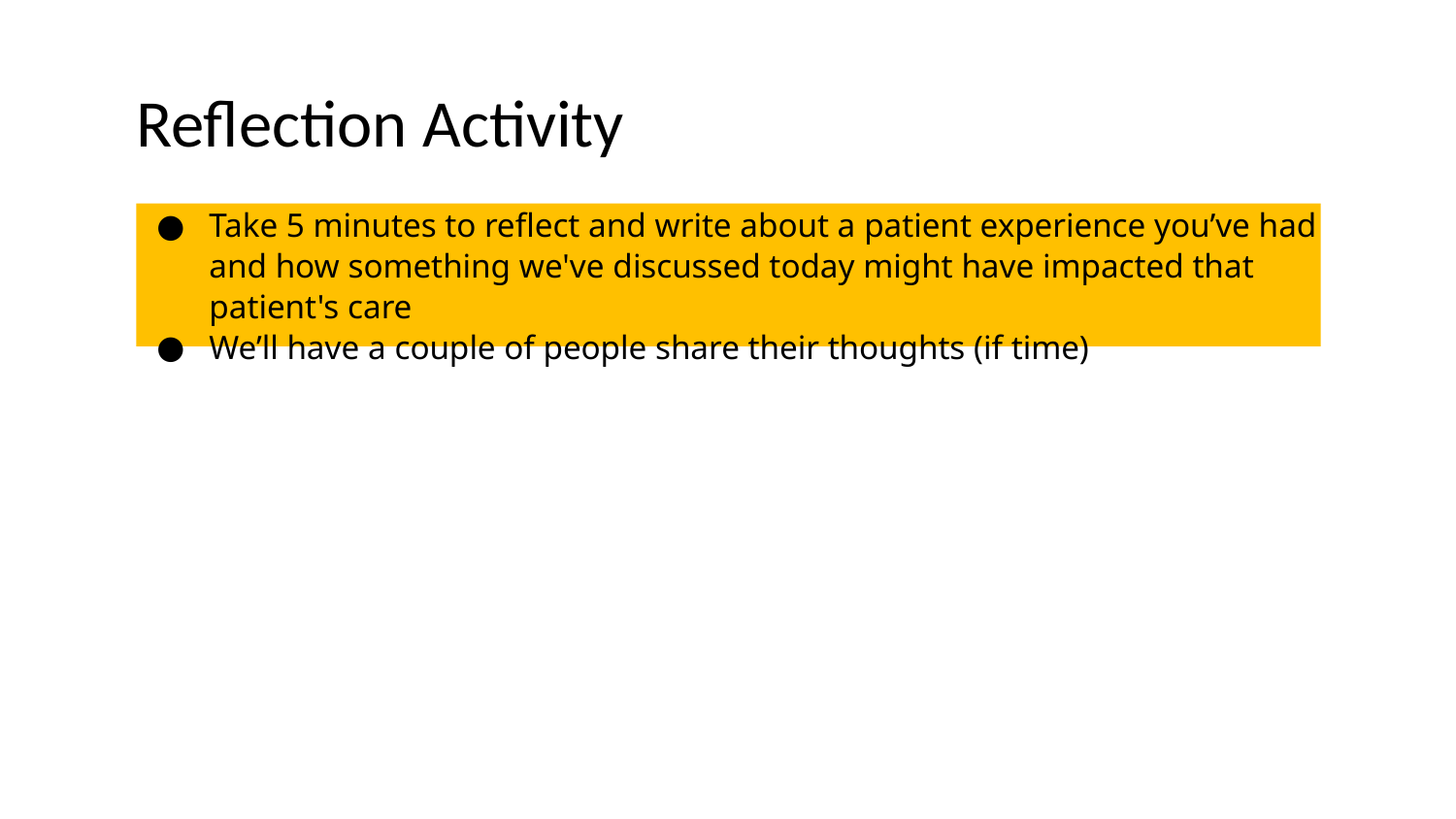

# Reflection Activity
Take 5 minutes to reflect and write about a patient experience you’ve had and how something we've discussed today might have impacted that patient's care
We’ll have a couple of people share their thoughts (if time)

## Slide 31
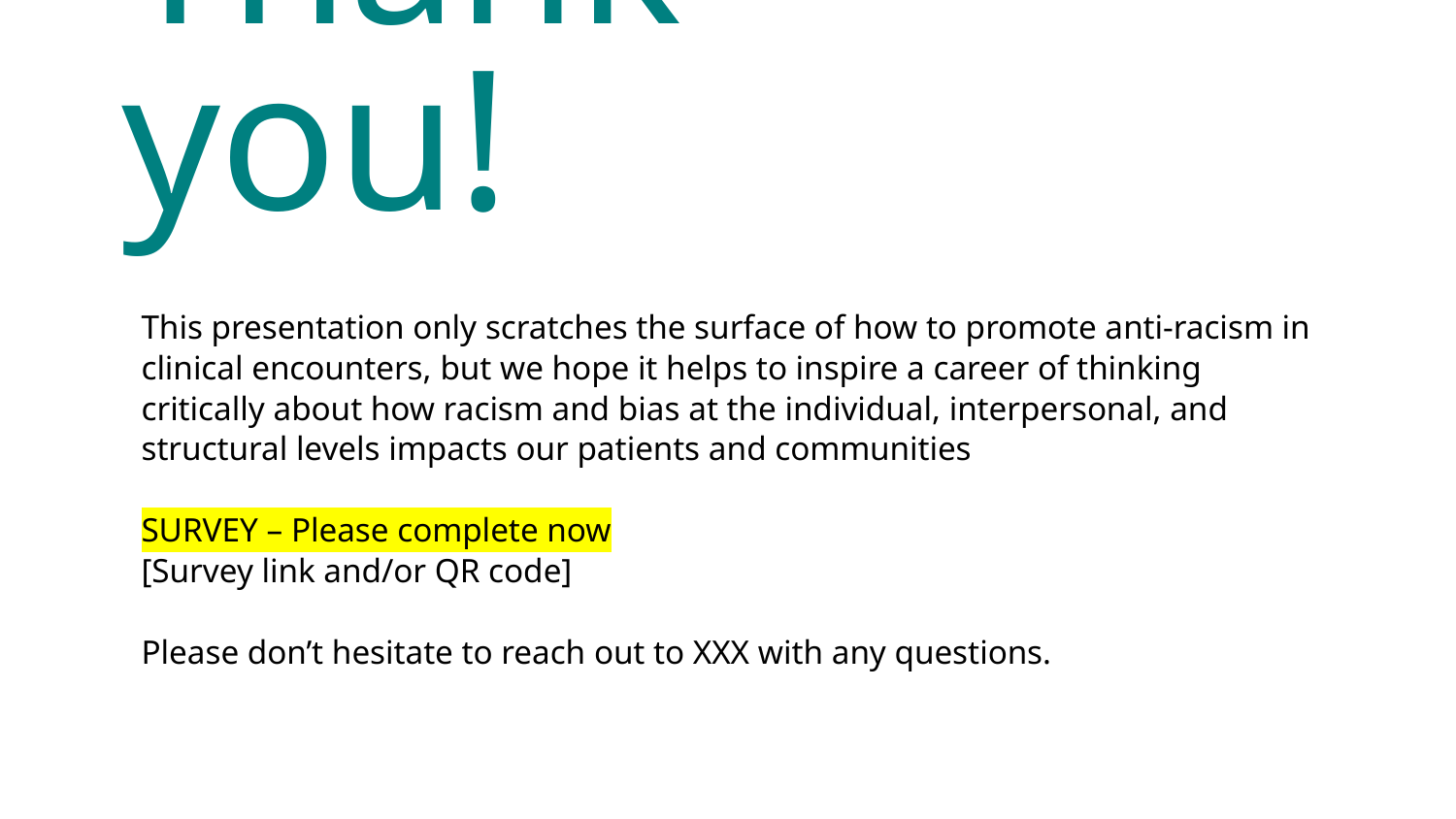

Thank you!
This presentation only scratches the surface of how to promote anti-racism in clinical encounters, but we hope it helps to inspire a career of thinking critically about how racism and bias at the individual, interpersonal, and structural levels impacts our patients and communities
SURVEY – Please complete now
[Survey link and/or QR code]
Please don’t hesitate to reach out to XXX with any questions.

## Slide 32
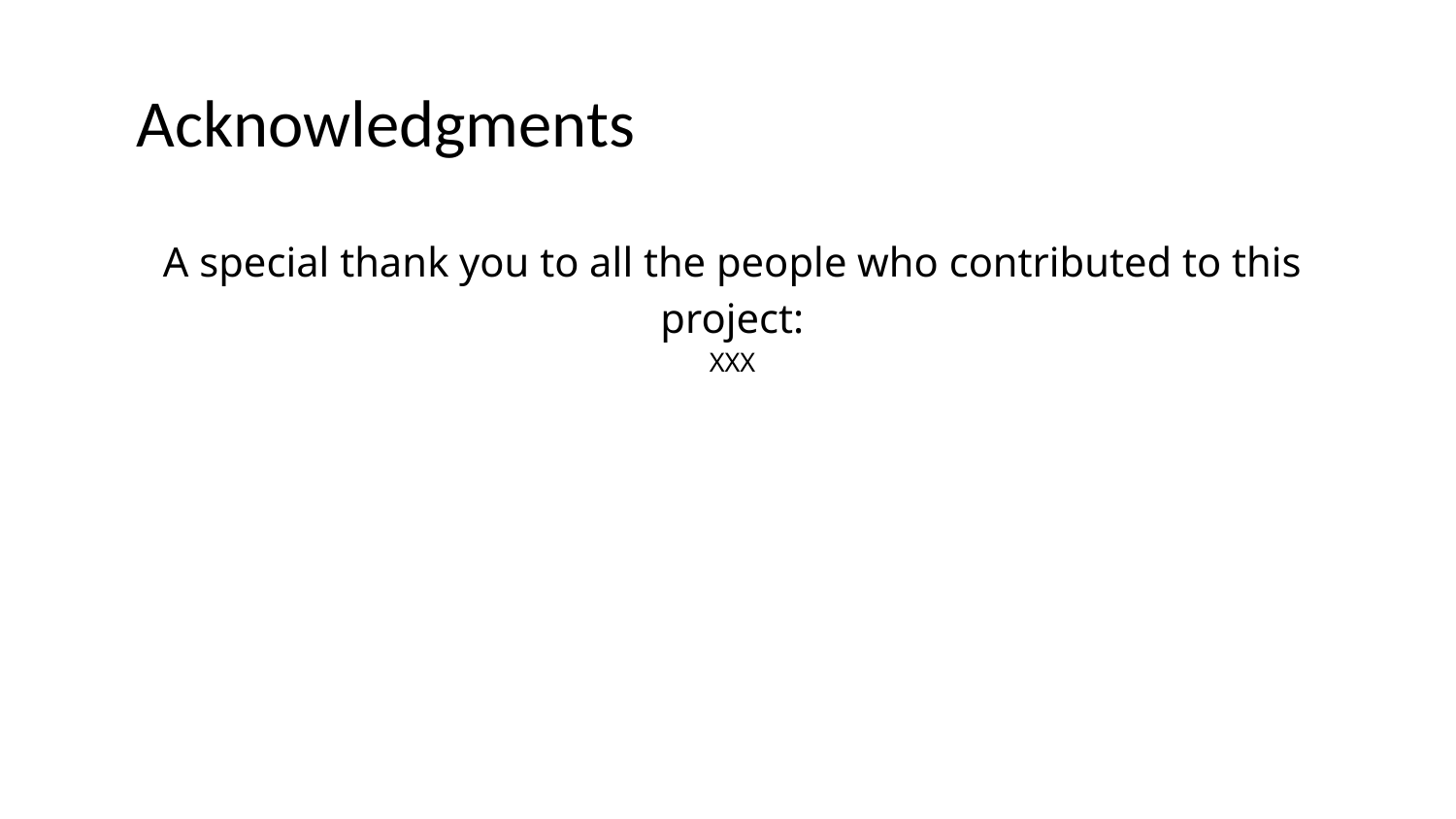

# Acknowledgments
A special thank you to all the people who contributed to this project:
XXX

## Slide 33
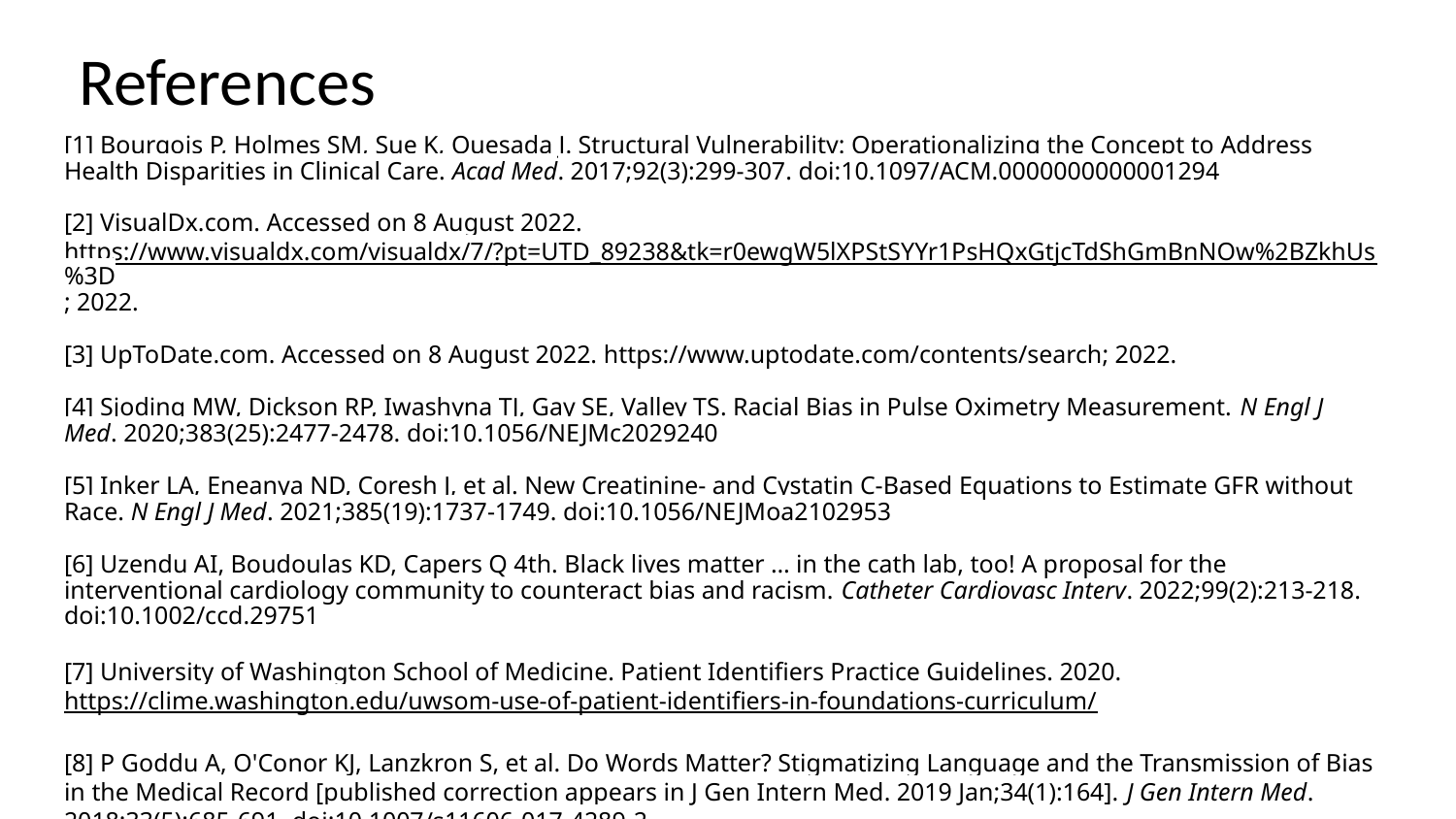

# References
[1] Bourgois P, Holmes SM, Sue K, Quesada J. Structural Vulnerability: Operationalizing the Concept to Address Health Disparities in Clinical Care. Acad Med. 2017;92(3):299-307. doi:10.1097/ACM.0000000000001294
[2] VisualDx.com. Accessed on 8 August 2022. https://www.visualdx.com/visualdx/7/?pt=UTD_89238&tk=r0ewgW5lXPStSYYr1PsHQxGtjcTdShGmBnNOw%2BZkhUs%3D; 2022.
[3] UpToDate.com. Accessed on 8 August 2022. https://www.uptodate.com/contents/search; 2022.
[4] Sjoding MW, Dickson RP, Iwashyna TJ, Gay SE, Valley TS. Racial Bias in Pulse Oximetry Measurement. N Engl J Med. 2020;383(25):2477-2478. doi:10.1056/NEJMc2029240
[5] Inker LA, Eneanya ND, Coresh J, et al. New Creatinine- and Cystatin C-Based Equations to Estimate GFR without Race. N Engl J Med. 2021;385(19):1737-1749. doi:10.1056/NEJMoa2102953
[6] Uzendu AI, Boudoulas KD, Capers Q 4th. Black lives matter … in the cath lab, too! A proposal for the interventional cardiology community to counteract bias and racism. Catheter Cardiovasc Interv. 2022;99(2):213-218. doi:10.1002/ccd.29751
[7] University of Washington School of Medicine. Patient Identifiers Practice Guidelines. 2020.  https://clime.washington.edu/uwsom-use-of-patient-identifiers-in-foundations-curriculum/
[8] P Goddu A, O'Conor KJ, Lanzkron S, et al. Do Words Matter? Stigmatizing Language and the Transmission of Bias in the Medical Record [published correction appears in J Gen Intern Med. 2019 Jan;34(1):164]. J Gen Intern Med. 2018;33(5):685-691. doi:10.1007/s11606-017-4289-2
